# Tracking Regulatory Mechanism of Trace Fe on Graphene Electromagnetic Wave Absorption

**DOI:** 10.1007/s40820-023-01280-6

**Published:** 2024-01-04

**Authors:** Kaili Zhang, Yuhao Liu, Yanan Liu, Yuefeng Yan, Guansheng Ma, Bo Zhong, Renchao Che, Xiaoxiao Huang

**Affiliations:** 1 School of Materials Science and Engineering, Harbin Institute of Technology, Harbin, 150001 People’s Republic of China; 2 MIIT Key Laboratory of Advanced Structural-Functional Integration Materials & Green Manufacturing Technology, Harbin Institute of Technology, Harbin, 150001 People’s Republic of China; 3 School of Materials Science and Engineering, Harbin Institute of Technology at Weihai, Weihai, 264209 People’s Republic of China; 4 Laboratory of Advanced Materials, Shanghai Key Lab of Molecular Catalysis and Innovative Materials, Fudan University, Shanghai, 200438 People’s Republic of China

**Keywords:** Reduced graphene oxide, Fe nanosheets, Dielectric loss, Electromagnetic wave absorption

## Abstract

Polarization and conductance losses are the fundamental dielectric attenuation mechanisms for graphene-based absorbers, but it is not fully understood in revealing the loss mechanism of affect graphene itself. For the first time, the reduced graphene oxide (RGO) based absorbers are developed with regulatory absorption properties and the absorption mechanism of RGO is mainly originated from the carrier injection behavior of trace metal Fe nanosheets on graphene. Accordingly, the minimum reflection loss (RL_min_) of Fe/RGO-2 composite reaches − 53.38 dB (2.45 mm), and the effective absorption bandwidth achieves 7.52 GHz (2.62 mm) with lower filling loading of 2 wt%. Using off-axis electron hologram testing combined with simulation calculation and carrier transport property experiments, we demonstrate here the carrier injection behavior from Fe to graphene at the interface and the induced charge accumulation and rearrangement, resulting in the increased interfacial and dipole polarization and the conductance loss. This work has confirmed that regulating the dielectric property of graphene itself by adding trace metals can not only ensure good impedance matching, but also fully exploit the dielectric loss ability of graphene at low filler content, which opens up an efficient way for designing lightweight absorbers and may be extended to other types materials.

**Supplementary Information:**

The online version contains supplementary material available at 10.1007/s40820-023-01280-6.

## Highlights

- A carrier injection strategy is firstly proposed by designing Fe/reduced graphene oxide (RGO) heterogeneous interfacial material for giving full play to the dielectric dispersion properties of graphene.
- The electromagnetic wave absorption mechanisms mainly include enhanced conductance loss, dipole polarization and interfacial polarization.
- Outstanding reflection loss value (− 53.38 dB, 2.45 mm) and broadband wave absorption (7.52 GHz with only 2 wt% filling) of Fe/RGO composite were acquired, which is superior to single-component graphene.

**Supplementary Information:**

The online version contains supplementary material available at 10.1007/s40820-023-01280-6.

## Graphical abstract

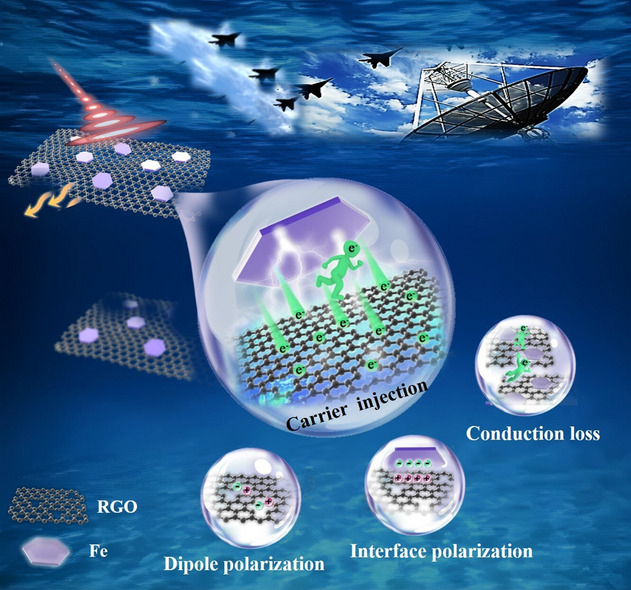

## Introduction

With the development of microwave technology and the popularization of electronic products, electromagnetic wave (EMW) not only brings great convenience to people’s life, but also produces a lot of electromagnetic (EM) interference [1–3]. EM radiation not only harms human health, but also affects the normal operation of electronic equipment. As the main barrier against EM interference, absorbing materials have been widely used in military and civil fields [4–8]. In recent years, carbon materials with thin thickness, low density and excellent electrical properties (such as graphite [9], graphene [10], carbon black [11] and carbon nanotubes (CNTs) [12]) have shown extensive application prospects in the EMW absorption field.

Graphene, a newly emerging carbon material with unique layered structure and intrinsic metallic conductivity, has emerged as promising candidates for EMW absorption [13–16]. Nevertheless, single-component graphene does not exhibit ideal EMW absorption ability due to the contradiction between impedance matching and high absorption strength. Previous studies have shown that designing chemical components and structural engineering of graphene are crucial for effectively improving the EMW absorption performance, particularly in combination with dielectric or magnetic components. For instance, the well-designed 3D accordion-like CeO_2−*x*_/RGO composites can effectively improve the impedance matching and display the minimum reflection loss (RL_min_) of − 50.6 dB at 15.9 GHz, and effective absorption bandwidth (EAB, RL ≤ − 10 dB) of 5.84 GHz with a filler content of 50 wt% [17]. Huang et al. [18] prepared the rGO–Fe_3_O_4_ absorbing material via hydrothermal reaction, amidation reaction and reduction process, and the EMW performance has been greatly improved due to the EM synergistic effect of rGO and Fe_3_O_4_. The resulting product exhibited the RL_min_ of − 48.6 dB at 14.4 GHz and EAB of 6.32 GHz with 45 wt% filler loading. Furthermore, there are still many metals coated on RGO (such as Fe/LrGO [19], GA@Ni [20], Co/GN [21] and rGO/FeCo [22]) to achieve a better impedance matching and attenuation. Despite their improved performance, the application and development still exist significant bottlenecks. On the one hand, the reported RGO-based absorbers have the high filler content disadvantage, which inevitably leads to impedance mismatch due to high conductivity of RGO. Although the impedance matching can be optimized by reducing the filling content, efficient attenuation cannot be achieved. This limits its practical application to some extent. In addition, there are few reports of graphene absorbers filling less than 5 wt%. On the other hand, although the EMW absorbing properties of graphene-based materials have been improved through component optimization and structural design with multiple loss mechanisms, it faces unclear problems about how graphene further exerts itself dielectric properties to enhance the dielectric attenuation mechanism in composites. Thus, it is urgent the effect mechanism of graphene to further understand dielectric properties, which has important application value for obtaining ideal graphene-based absorbing materials with low loading and high loss attenuation ability.

To explore the dielectric mechanism of graphene-based absorbers, the electron-hopping (EHP) model, aggregative-induced-charge-transport and conductive-network equation were first established by Cao’s groups to reveal the relationship between temperature, conductivity and dielectric behaviors of graphene layers [23–25]. This provides the theoretical basis for the dielectric loss of graphene. In addition, Fe/G nanocomposites exhibited obvious dielectric properties due to the interfacial coupling between graphene and metal and reached the bandwidth of 4.4 GHz with the thickness of 2.0 mm (40 wt%) [26]. It is reported that the interfacial interaction between various dopants and graphene (such as GfeTCs [27], GO/graphene [28], Ru/graphene [29], Ag/graphene [30]) would generate charge transfer, thus resulting in new electrical properties of graphene. Furthermore, the important polarization phenomenon between heterogeneous interfaces in graphene-based composites (such as Co/CoO/RGO [31], Fe_3_O_4_@NPC@rGO [32], Co@N-doped carbon [33] and Fe_3_O_4_–Fe_3_O_4_@C [34]) has been demonstrated by off-axis electron hologram and charge distribution map. These studies provide feasible strategies for designing graphene absorbers with excellent properties. However, there is still the problem of high absorbent filling rate due to the high density of the metal. Transition metal materials (Fe, Co, Ni) and their compounds have been considered as the popular candidates due to their intrinsic ferro-magnetic resonance absorption capacity in EMW absorption [35–38]. Moreover, due to its special atomic structural properties, metal Fe exhibits unique physical and chemical properties in many aspects. Special focus on iron oxide hematite (Fe_2_O_3_) is observed because it has the advantages of simple process, various methods, low price, high yield, good thermal stability and chemical stability [39]. Importantly, Fe_2_O_3_ is easily reduced to various morphologies of Fe. Metal Fe interior possesses abundant free electrons and the interfacial electronic interaction between metal and graphene would make graphene exhibit some novel electric properties [40]. Therefore, it is a challenge to make full use of the free electrons in metals to exert dielectric dispersion property of graphene to achieve excellent EMW absorption performance under low loading.

Herein, we introduced trace Fe nanosheets to construct novel Fe/reduced graphene oxide (Fe/RGO) composite absorber based on the different electron gain and loss capabilities (The work function of Fe is 4.5 eV and that of graphene is 5.0 eV) to gain insight into the regulatory effects of trace Fe on the dielectric properties of RGO. The results revealed that Fe/RGO exhibited strong dielectric dispersion characteristic and high conductivity compared with RGO. To clarify the mechanism of dielectric properties of RGO, Fe/graphene field effect transistor (FG-FET) device was fabricated using chemical vapor deposition and vacuum thermal evaporation methods to further explore the micro-interface electronic interaction between graphene and Fe. The results of the Hall effect and carrier transfer characteristic curves indicated that the interface between graphene and Fe is ohmic contact form, and free electrons of Fe are injected into graphene by transforming from graphene hole transport (*P*-type) to electron transport (*N*-type), which can be inferred that this process directly enhances the conduction loss. The charge accumulation and rearrangement resulting from the carrier injection process further increased interfacial polarization and dipole polarization thereby synergistically promoting dielectric attenuation performance, which has been verified by holography technology. Compared with RGO, the absorption performance of Fe/RGO-2 is greatly improved, with the RL_min_ value of − 53.38 dB and EAB of 7.52 GHz (10.48–18 GHz) at a lower filling loading of 2 wt%. This work not only provides new research evidence for the relationship between the electricity and dielectric properties of graphene, but also is expected to guide future exploration on designing high-performance graphene-based absorption materials.

## Experimental Section

### Preparation of Fe_2_O_3_ Nanosheets, Fe/RGO Composites and Fe/Graphene Field Effect Transistor (FG-FET)

#### Synthesis of Single-Crystal Hexagonal Fe_2_O_3_ Nanosheets

The α-Fe_2_O_3_ nanosheets were synthesized via a simple solvothermal method of FeCl_3_ in mixture solutions at suitable temperatures. The details are as follows. FeCl_3_·6H_2_O (1.10 g) was added into a mixture of ethanol (40.0 mL) and deionized water (2.8 mL) and stirred till solid powders were completely dissolved. With continuous magnetic stirring, CH_3_COONa (5.0 g) was added to the solution and stirred for 40 min. After the mixture was stabilized, the mixture was transferred to autoclave for solvothermal reaction at 180 °C for 18 h. The auburn product was washed by ethanol and distilled water for three times, respectively, and dried in a vacuum oven at 65 °C for 8 h. For the surface modification, hexagonal Fe_2_O_3_ nanosheets (0.6 g) and hexadecyltrimethylammonium bromide (CTAB, 0.2 g) were dispersed in deionized water (50 mL) with magnetic stirring in a 60 °C water bath for 2 h. The product was washed with deionized water for several times and dried at 65 °C, and finally the modified hexagonal Fe_2_O_3_ nanosheets were obtained.

#### Preparation of Fe/RGO Composites

First, GO was synthesized using a reported modified Hummers’ method [41]. Second, GO suspension (100 mL and 10 mg mL^−1^) were mixed with a certain amount of modified Fe_2_O_3_ (0, 2, 4, 6 and 8 mg), respectively, and the mixture was ultrasonic stirred evenly for 3 h by electrostatic adsorption process. Then, the mixtures were sealed and refrigerated in the refrigerator, and treated by freeze-drying method (− 65 °C, 0.1 Pa) for 24 h. The obtained precursors were named GO, Fe_2_O_3_/GO-1, Fe_2_O_3_/GO-2, Fe_2_O_3_/GO-3 and Fe_2_O_3_/GO-4, respectively. Finally, the above products were put into the tube furnace and heat treated at 350 °C for 2 h under H_2_ atmosphere (60 mL min^−1^). The samples were named RGO, Fe/RGO-1, Fe/RGO-2, Fe/RGO-3, Fe/RGO-4, respectively. Furthermore, the preparation of Co/RGO and Ni/RGO was consistent with the above methods (Supporting Information).

#### Preparation of Fe/Graphene Field Effect Transistor (FG-FET)

Monolayer graphene was grown using CH_4_ as carbon source, H_2_ as carrier gas and 25 μm thick Cu foil (111) as a growth matrix by chemical vapor deposition method [42]. The graphene film was transferred to SiO_2_/Si substrate by PMMA wet transfer process [43]. Then, Fe/graphene film were prepared by deposition of Fe (20 nm) on the surface of monolayer graphene. The Fe/graphene field effect transistor (FG-FET) was fabricated by thermal evaporation of Cr/Au (5/30 nm) electrodes on the surface of Fe/graphene films through a mask reticle to form source and drain electrodes. The preparation of graphene field effect transistor (G-FET) was consistent with the above method.

### Characterization

The micromorphology was investigated by scanning electron microscopy (SEM, SUPRA55, ZEISS MERLIN), and corresponding energy dispersive spectroscopy (EDS) mapping analyses were used for collecting elemental signals and mapping images. Transmission electron microscopy (TEM, Philips-FEI, Tecnai G2 F30) was carried out using Tecnai F30 electron microscope operating at 200 kV. The structure of as-synthesized materials was characterized by X-ray diffraction (XRD) collected on a DX-2700 X-ray diffractometer. Fourier transform infrared (FTIR, Nicolet is50) spectra were measured over the range of 4000–500 cm^−1^. The Raman spectra (532 nm) was collected with inVia-Reflex. The elemental composition of the sample surface was determined by X-ray photoelectron spectroscopy (XPS) measurements using a Thermal Escalab 250, with a monochromatic Al Kα X-ray source. The magnetic properties of hybrid materials were tested with a vibrating sample magnetometer (Quantum Design, Dynacool-14T). The composition content of the material was tested by ICP-MS (NexION 350X, PerkinElmer). The electrical properties of the materials were tested by scanning probe microscope (SPM, Bruker, Dimension Fastscan) with C-AFM, KPFM and EFM functions, and the conductive probe is Pt/Ir coated probes (Table S1). The electrical conductivity was measured by four probe methods (ST2742B). The current output curve of the composite was measured on the semiconductor characteristic system (Keithley 4200-SCS). The toroidal samples (*Φ*in:3.04 mm, *Φ*out:7.00 mm) were made of the hybrid material uniformly mixed with paraffin at 1–5 wt%. Its complex permittivity and permeability were characterized by using an Agilent N5230A vector network analyzer (VNA). The RL values were calculated based on the transmission-line theory as follows [44, 45]:

1 $${\text{RL}}\;\left( {{\text{dB}}} \right) = 20 \log \left| {\frac{{Z_{{{\text{in}}}} - Z_{0} }}{{Z_{{{\text{in}}}} + Z_{0} }}} \right|$$

2 $$Z_{{{\text{in}}}} = Z_{0} \sqrt {\frac{{\mu_{r} }}{{\varepsilon_{r} }}} \tanh \left( {j\frac{2\pi fd}{c}\sqrt {\mu_{r} \varepsilon_{r} } } \right)$$

where $Z_{{{\text{in}}}}$ and $Z_{0}$ represent the input impedance of the absorber and free space, *d* is the thickness of the sample, *c* is the velocity of light in free space.

### Calculation Details

All plane-wave within the density functional theory (DFT) calculations of atomic and electronic structures were performed using the CASTEP program provided in the Materials Studio 2020 package. The generalized gradient approximation (GGA) with Perdew–Burker–Ernzerhof (PBE) function was used to represent the exchange–correlation effects. The energy convergence criterion was 1 × 10^−5^ eV atom^−1^, the maximum force was 0.03 eV Å^−1^, the maximum stress was 0.05 GPa, and the energy cutoff was 400.0 eV. The Brillouin zone integration was sampled by using 2 × 2 × 1 k-point mesh density. TS method for DFT-D correction was used to accurately describe the van der Waals forces. The vacuum gap was set to be about 20 Å, which should lead to negligible interactions between the system and their mirror images.

## Results and Discussion

### Synthesis and Characterization of Fe/RGO Composites

**Fig. 1:**
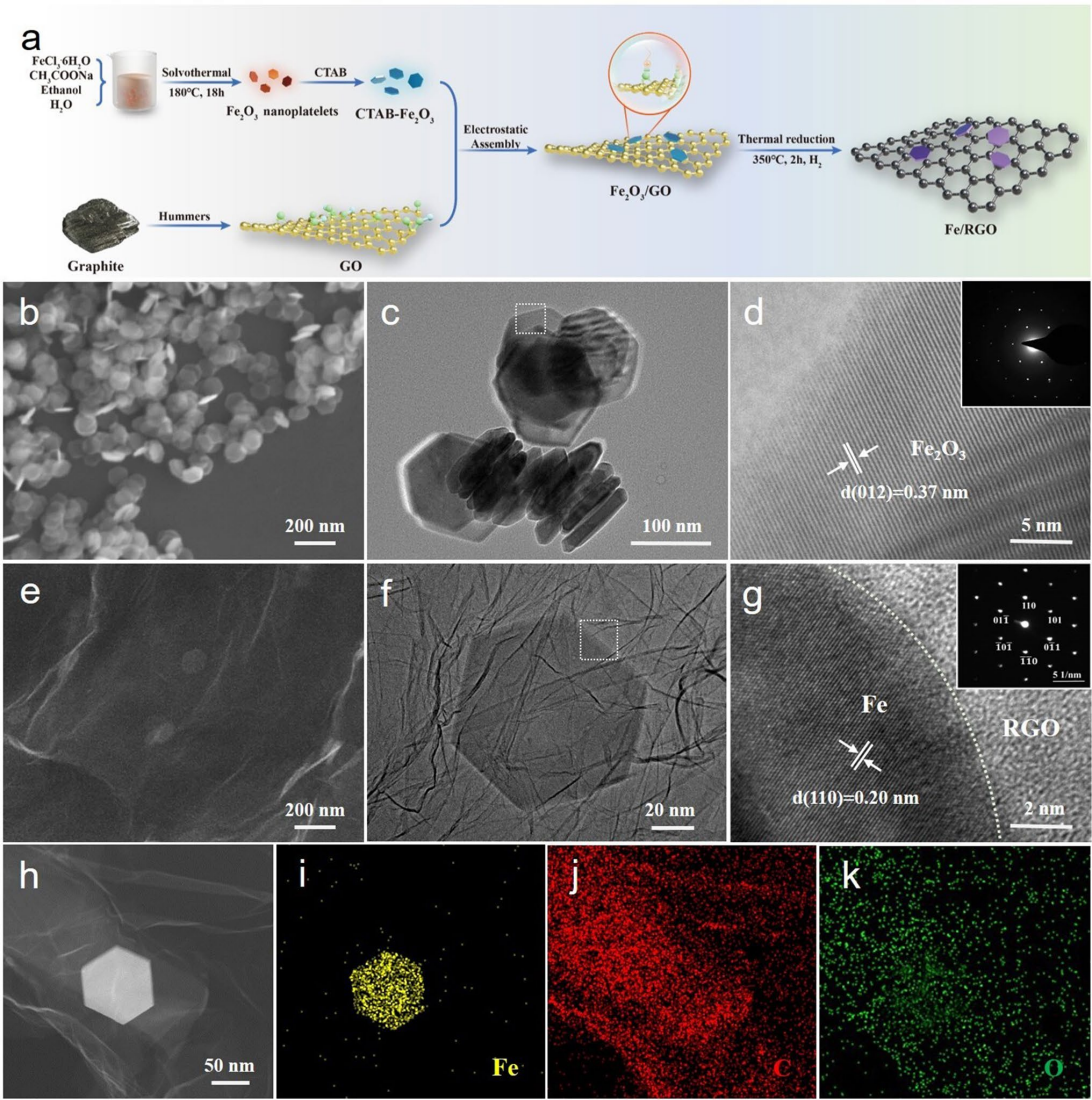
Synthetic process, microstructural characterization. **a** Schematic illustration of the preparation process of the Fe/RGO composites. **b** and **e** SEM images, **c**, **f** TEM images and **d**, **g** HRTEM images of Fe_2_O_3_ nanoplatelets and Fe/RGO-2 (Inset: the corresponding SAED pattern results). **h**–**k** STEM image and TEM-EDS map of Fe/RGO-2

Fe/RGO composites were synthesized via a facile electrostatic adsorption reaction and low temperature thermal reduction method (Fig. 1a). Firstly, Fe_2_O_3_ hexagonal nanosheets were synthesized by solvothermal method. And, GO (negatively charged) and modified Fe_2_O_3_ nanosheets (positively charged) were firmly combined by electrostatic adsorption method. Finally, Fe/RGO composite was prepared by freeze-drying and thermal reduction. By controlling the addition amount of modified Fe_2_O_3_ nanosheets (0, 2, 4, 6, and 8 mg), the effect of the content of Fe nanosheets on the electronic structure of RGO was investigated (labeled as RGO, Fe/RGO-1, Fe/RGO-2, Fe/RGO-3, and Fe/RGO-4). The microstructure of the composite was characterized by SEM/TEM. Fe_2_O_3_ presents a thin and uniformly distributed hexagonal nanoplate structure (Fig. 1b). It can be clearly seen that Fe_2_O_3_ crystallizes into a hexagonal nanosheet with a width of about 100 nm, a thickness of about 10 nm and an aspect ratio of about 0.1 (Fig. 1c). Moreover, as can be seen from the HRTEM image in Fig. 1d, the lattice spacing is 0.37 nm corresponding to the (120) crystal plane of α-Fe_2_O_3_ and the SAED pattern shows that the as-synthesized Fe_2_O_3_ nanosheet is single-crystalline. The morphology of Fe/RGO composite is shown in Fig. S1. The RGO sheets show translucent and abundant wrinkles which are conducive to the loading of Fe nanosheets. With the increase in Fe content, Fe nanosheets are obviously increased and evenly distributed on the RGO sheets. Meanwhile, we can also obtain the accurate content of Fe in Fe/RGO samples by ICP measurement, the percentage of Fe is 0.32, 0.68, 0.94, and 1.22 wt% in Fe/RGO-1, Fe/RGO-2, Fe/RGO-3, and Fe/RGO-4, respectively. Specifically, for the Fe/RGO-2 composite, Fe nanosheets are uniformly dispersed on the RGO sheets with wrinkles, showing a face-to-face contact pattern to increase the contact area as shown in Fig. 1e. In addition, TEM image shows that Fe maintains the hexagonal nanosheet microstructure (with a slightly reduced size) after thermal reduction (Fig. 1f). In order to investigate the Fe/RGO-2 in detail, the HRTEM and SAED images (Fig. 1g) are obtained from the Fe/RGO-2 edge. Fe with lattice spacing of 0.20 nm corresponding to the (110) crystal plane is monocrystalline, while RGO is amorphous carbon. The elemental distribution of Fe/RGO-2 is determined through the STEM image and TEM-EDS mapping in Fig. 1h–k, in which the C and O elements are uniformly distributed within the RGO sheets and Fe element shows a hexagonal distribution.

**Fig. 2:**
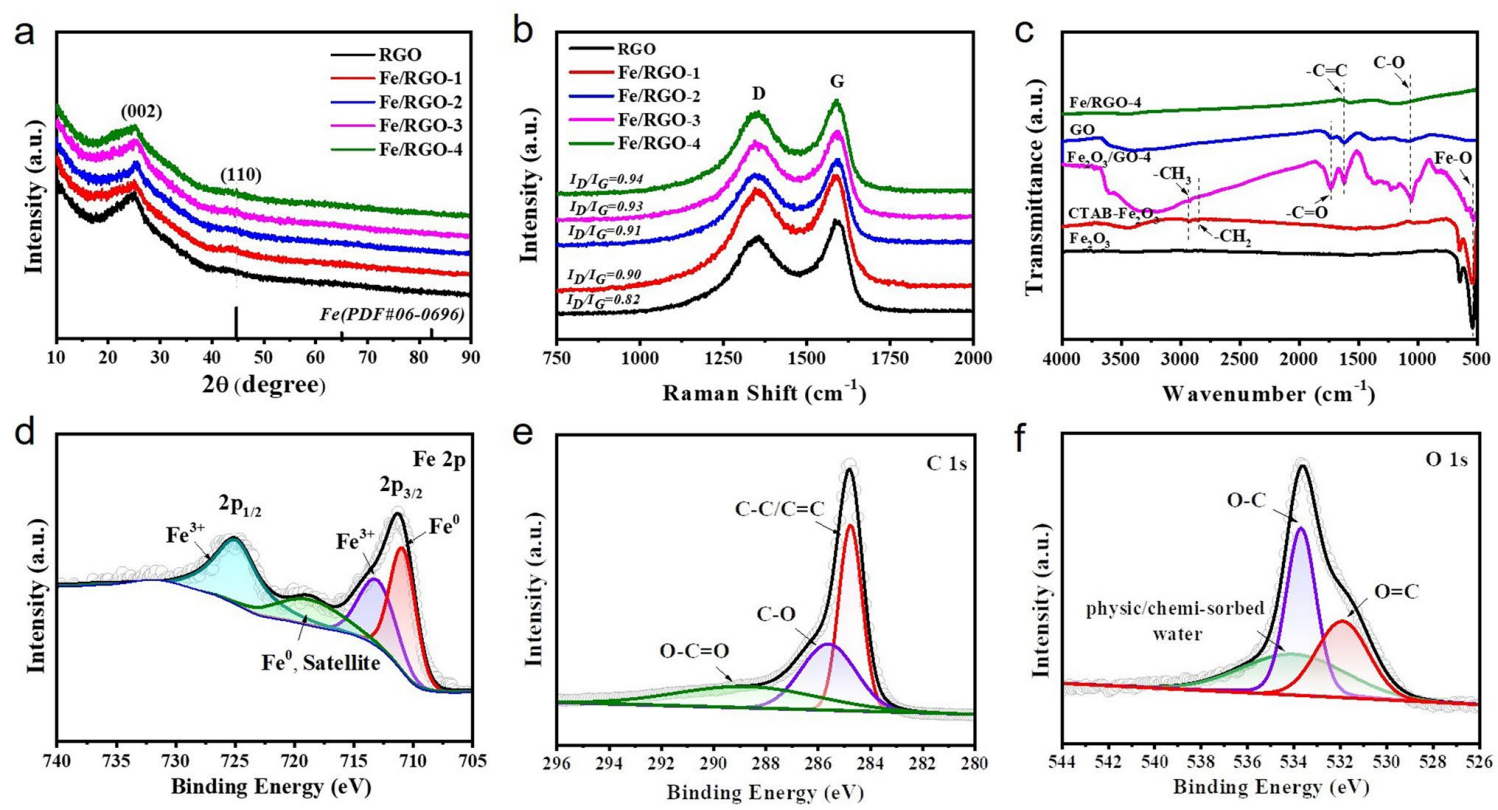
Structure characterization of Fe/RGO composites. **a** XRD patterns and **b** Raman spectra of RGO, Fe/RGO-1, Fe/RGO-2, Fe/RGO-3 and Fe/RGO-4. **c** FTIR spectra of Fe_2_O_3_, CTAB-Fe_2_O_3_, GO, Fe_2_O_3_/GO-4 and Fe/RGO-4, respectively. **d**–**f** XPS Fe 2*p* spectra, C 1*s* spectra and O 1*s* spectra of Fe/RGO-4

The crystal structure of the samples is characterized by XRD pattern. From Fig. 2a, all samples show amorphous carbon with a wide diffraction peak at around 26°, consisted with the result of HRTEM image (Fig. 1g). The typical diffraction peak of GO disappeared at 9.8°, indicating the GO was successfully reduced (Fig. S2a). In addition, compared with the strong diffraction peaks of pure Fe_2_O_3_ and Fe, the characteristic diffraction peaks of Fe_2_O_3_ and Fe nanosheets in Fe_2_O_3_/GO and Fe/RGO samples are not obvious, which is attributed to the minimal addition content of Fe_2_O_3_ (Fig. S2b). The samples were further analyzed by Raman spectrum. The three characteristic peaks correspond to the D band, G band and 2D band in Figs. 2b and S2d. The D band peak (1351 cm^−1^) and the G band peak (1590 cm^−1^) represent defects and disorder of graphitic carbon, and 2D band is related to the degree of graphene stacking. The intensity ratio (*I*_D_/*I*_G_) of the D and G band peaks reflects the degree of in-plane and edge defects of carbon skeleton [46]. As shown in Fig. 2b, the *I*_D_/*I*_G_ ratios of RGO, Fe/RGO-1, Fe/RGO-2, Fe/RGO-3, and Fe/RGO-4 are 0.82, 0.90, 0.92, 0.93, and 0.94, respectively, indicating that the introduction of Fe is unfavorable to the reconstruction of carbonization. But more defects will lead to more dipole polarization, which is beneficial for dielectric loss. In this work, GO was reduced to RGO by thermal reduction method. At low temperature (350 °C), the degree of RGO lamellar stripping is lower and there is stack, resulting in weak 2D peaks (Fig. S2d). Figure 2c shows the FTIR spectrum of Fe_2_O_3_, CTAB-Fe_2_O_3_, GO, Fe_2_O_3_/GO-4 and Fe/RGO-4. The absorption peak of 536 cm^−1^ is attributed to the Fe–O stretching vibration in Fe_2_O_3_, proving that Fe_2_O_3_ has been successfully prepared [47]. CTAB was successfully grafted onto the Fe_2_O_3_ surface due to the presence of –CH_3_ and –CH_2_ stretching vibration peaks at 2930 and 2846 cm^−1^ [48]. Compared with GO and Fe_2_O_3_/GO-4, Fe/RGO-4 shows C=C and C–O stretching vibration peaks at 1623 and 1060 cm^−1^, indicating that its oxygen-containing functional groups are significantly reduced after thermal reduction. The electronic structure and composition of the Fe/RGO-4 were characterized via XPS as shown in Fig. 2d–f. The curve-fitting of high-resolution Fe 2*p* spectrum observes the two typical peaks at 711.2 and 724.8 eV, assigning to Fe 2*p*_3/2_ and Fe 2*p*_1/2_ (Fig. 2d). And the two peaks at 710.8 and 718.9 eV of Fe 2*p* spectrum reflect the existence of the metallic state of Fe^0^, which indicates the formation of α-Fe after thermal reduction. The characteristic peaks correspond to the Fe^3+^ 2*p*_3/2_ and 2*p*_1/2_ at 713.2 and 725.0 eV and the appearance of Fe^3+^ could be ascribed to the oxidation of Fe on the surface of the material during testing [49]. The C 1*s* spectra of Fe/RGO-4 in Fig. 2e display the three peaks at 284.8, 286.0 and 289.7 eV, corresponding to the C=C/C–C, C–O and C=O, respectively. Moreover, the O 1*s* spectra of Fe/RGO-4 show the peaks at 531.9, 532.8 and 534 eV, corresponding to O=C, O–C and the adsorbed water via physic/chemistry method on the surface (Fig. 2f) [50]. Compared with the GO (Fig. S3), most oxygen-containing functional groups of Fe/RGO-4 were removed during the thermal reduction process.

### Analysis of EMW Absorption Performance and Loss Mechanism of Fe/RGO

**Fig. 3:**
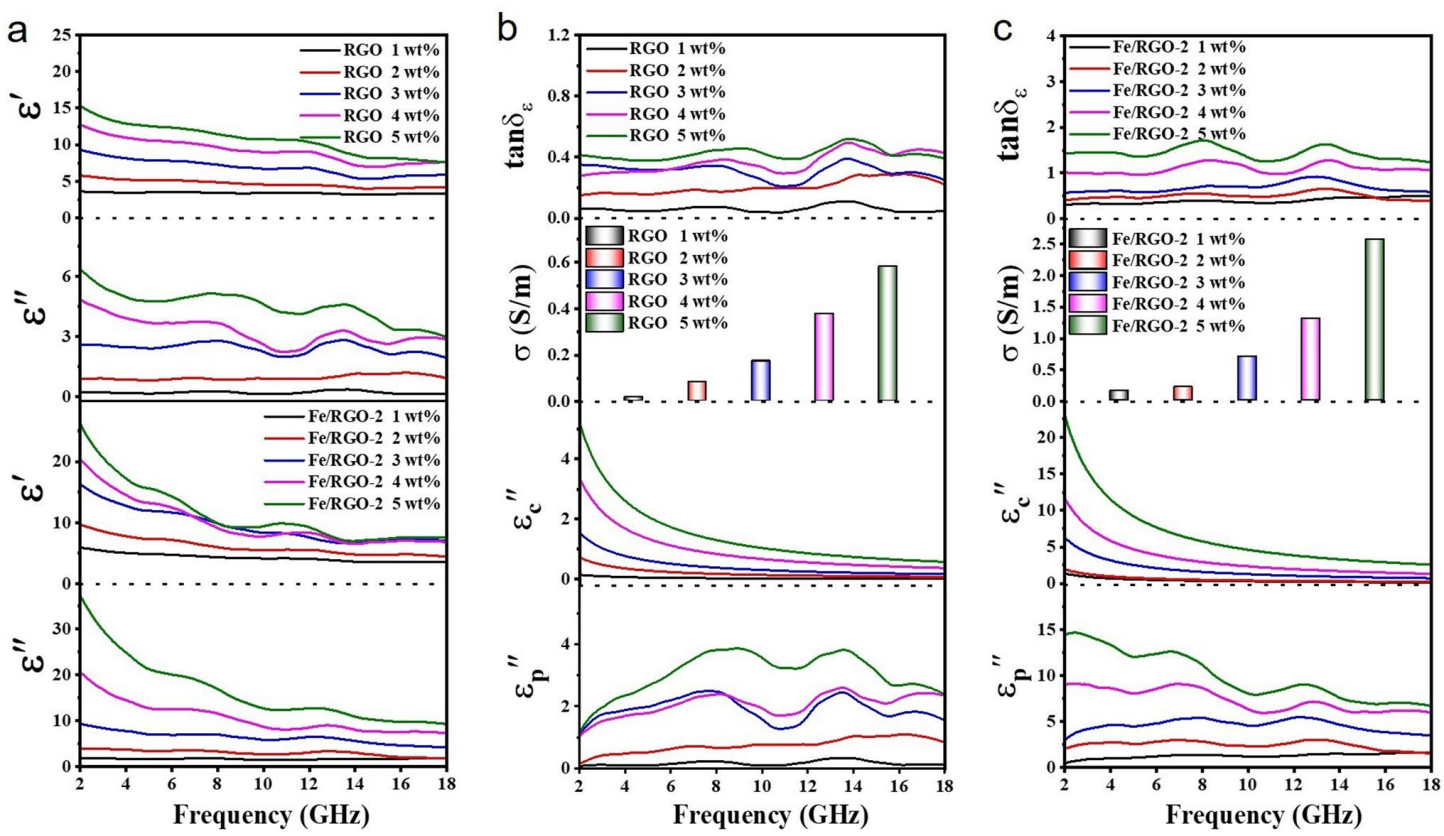
Analysis on EMW absorption mechanisms of RGO and Fe/RGO-2 composites. **a** *ε*′ and *ε*″. tan*δ*_*ε*_, *σ*, *ε*_c_″ and *ε*_p_″ of **b** RGO and **c** Fe/RGO-2 composites with 1–5 wt% loading

In general, the absorption properties of EMW are closely related to the complex permittivity (*ɛ*_*r*_ = *ɛ*′ − j *ɛ*″) and complex permeability (*μ*_*r*_ = *μ*′ − *j* *μ*″). The real part (*ɛ*′ and *μ*′) can be represented as the storage capacity of electrical and magnetic energy, while the imaginary part (*ɛ*″ and *μ*″) represents the loss capacity, respectively. The tangent of dielectric loss and magnetic loss (tan*δ*_*ε*_ and tan*δ*_*μ*_) represent the degree of EM energy loss capacity [51–53]. In order to explore the absorption mechanism of EMW, the EM parameters of RGO and Fe/RGO-2 were measured by VNA with 1–5 wt% filler loading as shown in Fig. 3. Figure 3a shows the variation of *ε*′ and *ε*″ curves of RGO and Fe/RGO-2 composites with the frequency of the incident EMW. RGO has the lowest value of the *ε*′ and *ε*″ at the low addition of 1 wt%, and the values of both the *ε*′ and *ε*″ show an increasing trend with the increase in the filling ratio, and a decreasing trend with the increase in frequency, showing dielectric dispersion characteristics. This is mainly to establish a strong conductive network structure. Interestingly, compared with RGO, Fe/RGO-2 exhibits higher *ε*′ and *ε*″ of dielectric constant and more obvious dispersion characteristic at the same filling content. The *u*′ and *u*″ of the permeability of RGO and Fe/RGO-2 are 1 and 0 without magnetic loss in Fig. S4. The absorption performance of samples were calculated by the RL value based on the transmission-line theory (Fig. S5). The RGO composite gradually shows significant absorption performance, while Fe/RGO-2 performance is poor with the increase in filling ratio. Surprisingly, compared to RGO, Fe/RGO-2 shows superior absorption performance with RL_min_ value reaching − 46.71 dB at 14.39 GHz (2.5 mm) and EAB of 6.73 GHz (11.27–18 GHz) in 2% filler loading. Under 4 wt%, the EAB of RGO reaches 4.84 GHz (11.42–16.26 GHz) at a thickness of 2.0 mm and the RL_min_ of − 36.96 dB is achieved at 8.59 GHz (3.0 mm), while Fe/RGO-2 has no effective absorption. Compared with RGO, the attenuation constants (*α*) of Fe/RGO-2 composites increase significantly, indicating the obvious improvement of absorption performance by the addition of Fe for RGO, while the variation trend of the impedance matching ratio (*Z*) of Fe/RGO-2 is opposite (Fig. S6). Therefore, to achieve high efficient EMW absorption, both α and *Z* should be considered. In order to understand the reason for the obvious change in the absorption performance of RGO and Fe/RGO, the dielectric loss mechanism are further analyzed, as shown in Fig. 3b, c. Due to the addition of low content (0.68 wt%) of Fe, the dielectric loss capacity (tan δ_*ε*_) and conductivity (*σ*) of Fe/RGO-2 is more significant and increase with the increase in filling ratio. Based on the Debye relaxation theory and Cole–Cole model, the polarization and conductivity loss play important roles in the changing of permittivity [46]. The *ε*′ and ε″ can be expressed as follows [54, 55]:

3 $$\varepsilon_{r} = \varepsilon_{\infty } + \frac{{\varepsilon_{s} - \varepsilon_{\infty } }}{{1 + i\left( {2\pi f} \right)\tau }} + \frac{\sigma }{{i\left( {2\pi f} \right)\varepsilon_{0} }}$$

4 $$\varepsilon ^{\prime} = \frac{{(\varepsilon_{s} - \varepsilon_{\infty } )}}{{1 + \left( {2\pi f} \right)^{2} \tau^{2} }} + \varepsilon_{\infty }$$

5 $$\varepsilon^{\prime \prime } = \varepsilon_{{\text{p}}}^{\prime \prime } + \varepsilon_{{\text{c}}}^{\prime \prime } = \frac{{\varepsilon_{s} - \varepsilon_{\infty } }}{{1 + \left( {2\pi f} \right)^{2} \tau^{2} }}2\pi f\tau + \frac{\sigma }{{2\pi f\varepsilon_{0} }}$$

The *ε*_c_″ and *ε*_p_″ curves represent the contribution of conductance loss and polarization loss to *ε*″, which is mainly conductance loss at low frequency and polarization loss at high frequency. It is inferred that the Fe nanosheets could enhance the polarization behavior and dielectric loss ability of RGO.

### Electronic Transport Mechanism of Fe/Graphene

**Fig. 4:**
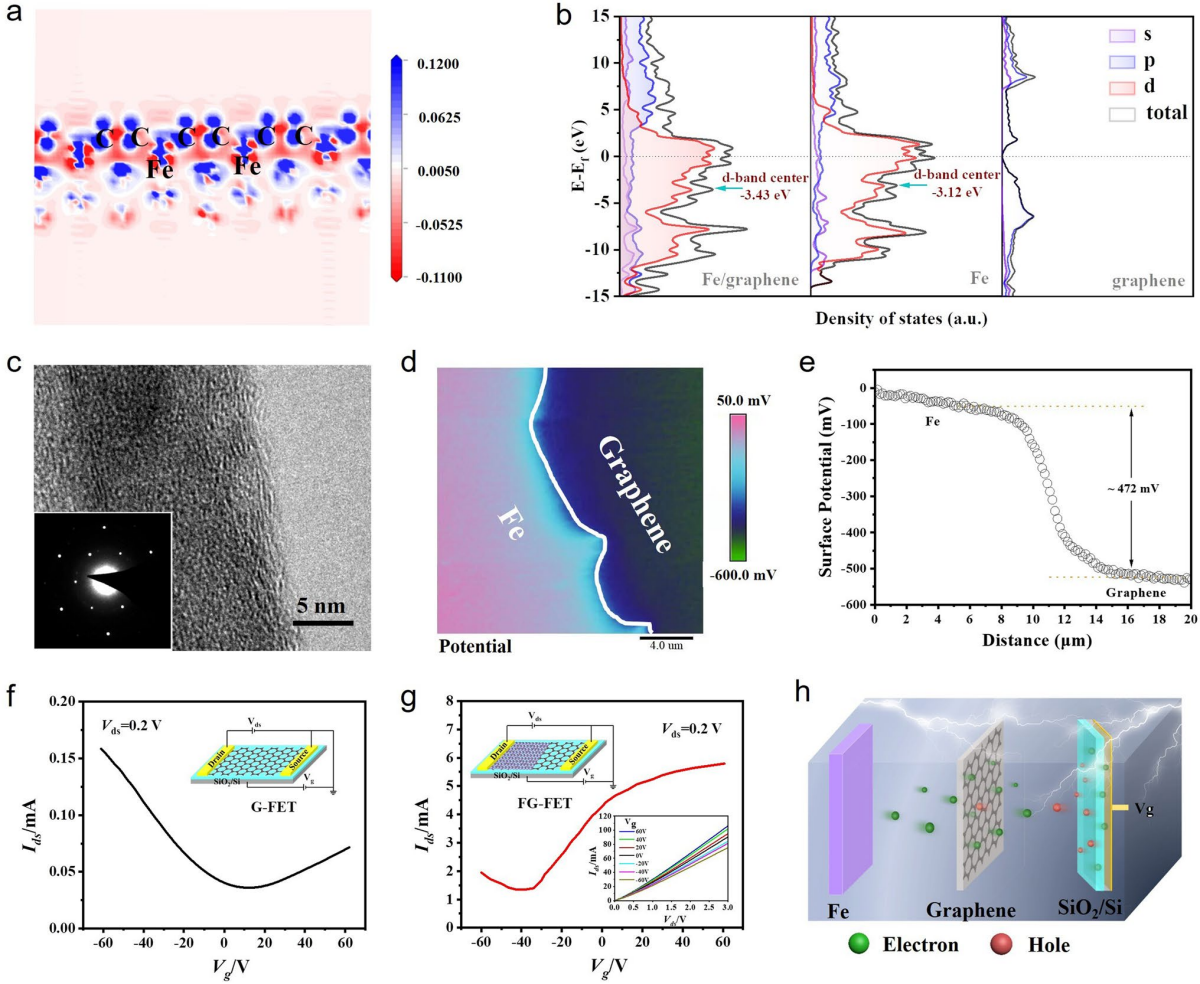
Electrical performance of the Fe/graphene. **a** Charge density difference plots of monolayer graphene adsorbed on Fe (110). The positive and negative charges are shown in blue and red (isovalue: 0.1). **b** DOS and PDOS results of Fe/graphene, Fe and graphene. **c** HRTEM image and SAED pattern of graphene. **d** Surface potential image and **e** the corresponding contact potential difference marked with white line of Fe/graphene. The transfer characteristic curves of **f** G-FET and **g** FG-FET at drain bias of 0.2 V (the insets show schematics of models and output characteristics at variable gate voltage). **h** Schematic diagram of working principle of *N*-type doping effect in FG-FET channel layer

In order to understand the effect mechanism of metal Fe on graphene electrical property in detail, the optimized geometric structure of Fe/graphene was constructed using perfect monolayer graphene as shown in Fig. S7. The charge density difference, electron localization function (ELF) and density of states (DOS) of Fe (110)/graphene (001) are calculated by DFT to analyze the electronic interaction between Fe and graphene. The charge accumulation (blue region) on graphene surface and charge depletion (red region) around Fe surface prove the close electronic interaction between Fe and graphene. Meanwhile, the detailed electron transfer within Fe/graphene can be reflected by isosurfaces obtained from ELF and Bader charge (Fig. 4a). The results indicate that 4.03 electrons are transferred from Fe atoms to C atoms and the interfacial charge distribution is mainly confined to the interface between graphene and Fe. Moreover, the density of states (DOS) and partial density of states PDOS of Fe/graphene, graphene and Fe are shown in Fig. 4b. The bandgap of graphene monolayer is 0 eV, and the valence and conduction bands are mainly contributed by the 2*p* orbital of C atom. Compared with Fe, the d-band center of the Fe/graphene moves to a low level around the Fermi level, indicating the charge transfer between Fe and C atoms. This is mainly due to the strong hybridization in Fe-3*d* and C-2*p* orbitals after the introduction of Fe atoms, which promotes the effective electron transport at the interface to achieve graphene carrier injection. From the theoretical calculation results, it can be concluded that Fe can stably combine with graphene by tight electronic interactions, and improve the electrical performance of graphene by changing the electronic states. To further verify the electron transport behavior of Fe/graphene structural model, FG-FET was constructed to explore the carrier injection type and contact mode of Fe/graphene by CVD and vacuum thermal evaporation methods. From HRTEM, SAED and Raman images, it can be shown that the growing graphene is monolayer and single crystal, and Fe is polycrystalline under experimental conditions (Figs. 4c and S8). First of all, the surface potential of Fe in contact with graphene and graphene was analyzed to evaluate the barrier height using KPFM. From Fig. 4d, it was obviously observed that the contact potential difference change between Fe and graphene layers, and inferred that the barrier height is about 472 mV, which proves that there is effective charge transfer between Fe and graphene (Fig. 4e). Subsequently, the Fe/graphene interface carrier transport behavior was characterized at room temperature by constructing FET. From Fig. 4f, g, the G-FET shows typical *P*-type (hole) carrier-dominated transfer characteristic behavior and FG-FET shows typical *N*-type (electron). Meanwhile, the Hall coefficient (RH) of graphene and Fe/graphene also verified the change of carrier type by Hall effect measurement (RH(graphene) = 6.03 × 10^−7^ m^3^/C, RH(Fe/graphene) = − 4.36 × 10^−6^ m^3^/C). Moreover, the output characteristic curves of the FG-FET show that the output current increases continuously with the increase in the positive gate voltage and presents an obvious linear relationship, indicating that the carrier transport type is mainly electron and a good ohmic contact is formed between the graphene and the Fe interface. According to the free electron theory of metals [56], free electrons inside Fe (as an electron donor) can form directional transport to graphene (as an electron acceptor) in the external electric field (Fig. 4h). Therefore, it can be inferred that the ohmic contact form formed by Fe/graphene can better promote the electron transport of Fe to graphene to achieve carrier injection, thus improving the electrical properties of graphene.

### EM Parameters Analysis and EMW Absorption Ability

**Fig. 5:**
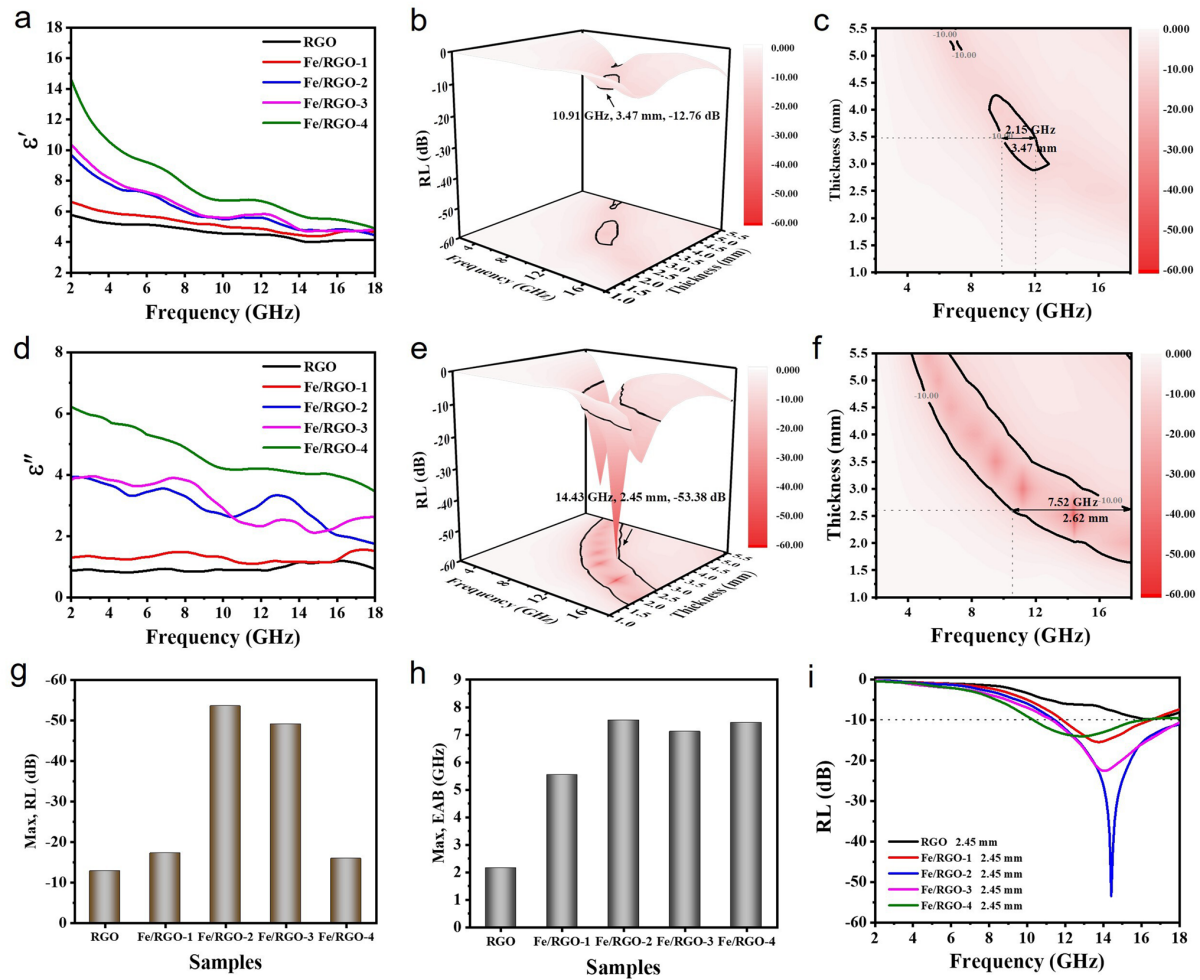
Analysis on EMW absorption performance of Fe/RGO composites with 2 wt% loading. **a**, **d** *ε*′ and *ε*″ of permeability. 3D and 2D color-mapping of calculated theoretical RL values with different thickness of **b**, **e** RGO and **e**, **f** Fe/RGO-2. Summarizations of **g** maximum RL and **h** EAB at different thickness for RGO, Fe/RGO-1, Fe/RGO-2, Fe/RGO-3, and Fe/RGO-4 composite samples. **i** RL value versus frequency for specific thickness values of samples

To further clarify the EMW absorption mechanism of Fe/RGO, the dielectric properties of Fe/RGO composites containing different Fe contents are studied with the filler loading of 2 wt% as shown in Fig. 5. In general, the *ε*′ and *ε*″ curves of RGO, Fe/RGO-1, Fe/RGO-2, Fe/RGO-3 and Fe/RGO-4 composites show an increasing trend with the increase in Fe content, and a decreasing trend with the increase in frequency, which has an obvious dielectric dispersion behavior (Fig. 5a, d). At 2 GHz, the *ε*′ value increase from 5.78 to 14.56 and *ε*″ increase from 0.88 to 6.21. Metallic Fe possesses good electrical conductivity and abundant free electrons inside according to the free electron theory [57]. Electrons are transferred to RGO more quickly along the interface of Fe/RGO in the form of ohmic contact, which plays a crucial role in increasing the imaginary part of the complex permittivity. In addition, the introduction of Fe nanosheets also leads to stronger polarization. Fe/RGO-2 and Fe/RGO-3 show significant *ε*″ resonance fluctuations around 8 and 13 GHz, which may be due to the enhanced interfacial polarization. Due to the low Fe content (0.32–1.22 wt%) in Fe/RGO composites, the *u*′ and *u*″ values of all samples are ≈ 1 and ≈ 0 (Fig. S9). The absorbing properties of Fe/RGO composites change significantly with the difference of Fe content. Figures 5b-f and S10 describe the EMW absorption performances of RGO, Fe/RGO-1, Fe/RGO-2, Fe/RGO-3, and Fe/RGO-4 composites with the thickness of the samples varying from 1 to 5.5 mm at 2–18 GHz. The RL_min_ of RGO is − 12.76 dB at 10.91 GHz (3.47 mm). In contrast, the strongest RL_min_ of Fe/RGO-1, Fe/RGO-2, Fe/RGO-3, and Fe/RGO-4 are − 17.08, − 53.38, − 49.57, and − 15.86 dB at 4.31, 2.45, 3.04 and 2.02 mm, respectively, which are higher than those of RGO (Fig. 5g). Compared with RL_min_, the EAB is more convincing for the absorption performance (the corresponding color image map). For the RGO, the maximum EAB is 2.15 GHz (9.92–12.07 GHz). In detail, the maximum EAB of Fe/RGO-2, Fe/RGO-3, and Fe/RGO-4 achieves the full *Ku* band absorption, which is 7.52 GHz (10.48–18 GHz), 7.03 GHz (10.97–18 GHz), and 7.45 GHz (10.55–18 GHz) corresponding to the thickness of 2.62, 2.49, and 2.41 mm, respectively (Fig. 5h). In particular, Fe/RGO-2 and Fe/RGO-3 composites exhibit high loss strength and wide-band absorption properties compared to other samples, which can be attributed to the synergistic dielectric polarization effect of Fe and RGO in the system. Figure 5i depicts the RL variation curves of RGO, Fe/RGO-1, Fe/RGO-2, Fe/RGO-3, and Fe/RGO-4 at 2.45 mm. At the same thickness, the EMW absorption performance of Fe/RGO shows a trend of increasing first and then decreasing, and moves to low frequency with the increase in Fe content. Compared with RGO, Fe/RGO-2 and Fe/RGO-3 have better EMW absorption performance (Fig. S11). It should be pointed out that the optimal RL_min_ peaks are shifted toward lower frequencies with increasing sample thickness which could be expressed by the quarter-wavelength cancelation law [58]:

6 $$t_{{\text{m}}} = \frac{n}{4}\lambda = \frac{nc}{{4f_{{\text{m}}} \sqrt {\left| {\mu_{r} \varepsilon_{r} } \right|} }}\quad n = 1, 3, 5, \ldots$$

where $t_{{\text{m}}}$ is the matching thickness, $\lambda$ represent the wavelength of the EMW, $t_{{\text{m}}}^{\exp }$ is the actual absorber thickness and $f_{{\text{m}}}$ is the matching frequency. It is obvious that the $t_{{\text{m}}}$ values of Fe/RGO composites are completely consistent with the curve of quarter-wavelength attenuation law. When the matching thickness of the sample satisfies formula (6), incident wave will reflect 180° on each interface with inverse phases, leading to energy attenuation of EMW. In addition, impedance matching properties should be demanded for an ideal EMW absorber. The EMW absorber with well-matched impedance can decrease the reflection of the EMW and facilitate the absorption. The impedance match value ($\left| {Z_{{{\text{in}}}} /Z_{0} } \right|$) approaching 1 indicates that the great mass of EMWs enter the absorber to attenuate [59]. It can be seen that the impedance matching of RGO at different thicknesses is far away from 1 to varying degrees, indicating that its impedance matching is poor and the RL_min_ value is also worse. The impedance matching values of Fe/RGO-2 and Fe/RGO-3 at different thicknesses are very close to 1. The best impedance matching degree and RL_min_ were corresponding to the *λ*/4, implying that the preferable performance is due to the outstanding impedance matching.

### Analysis of EMW Absorption Mechanism

**Fig. 6:**
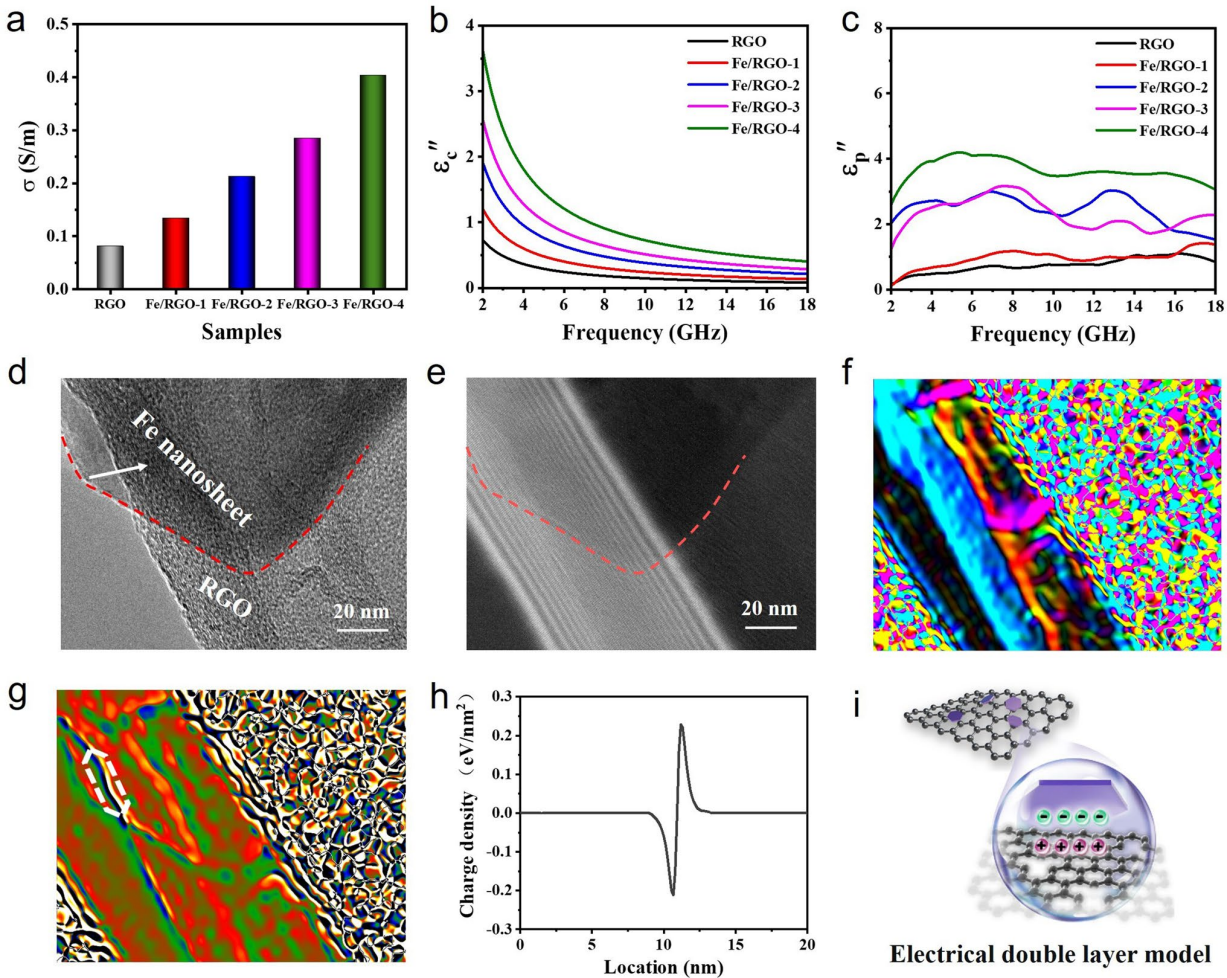
Analysis on EMW absorption mechanisms of Fe/RGO composites. **a** *σ*, **b** *ε*_c_″, **c** *ε*_p_″ of RGO, Fe/RGO-1, Fe/RGO-2, Fe/RGO-3, and Fe/RGO-4. **d**, **e** TEM and corresponding off-axis electron holography image, **f** Electric field distribution, **g** Charge density map, **h** Charge density profile along the white region, **i** The electrical double layer model of Fe/RGO

In order to analyze the effect of Fe content on the EMW absorption performance of RGO, the dielectric loss mechanisms of Fe/RGO are studied, as shown in Fig. 6. The free electrons inside the metallic Fe are transferred along the Fe/RGO interface to the RGO in the form of migration and hopping, which play a crucial role in increasing conductivity due to the carrier injection mechanism, as shown in Fig. 6a. And the conductance loss is the main factor and decreases with the increase in frequency at low frequency, while the polarization loss plays a leading role at high frequency (Fig. 6b, c). With the increase in Fe, the additional injected electrons at the heterogeneous interface increase the conduction loss (*ε*_c_″) in the EM field. The high *ε*_p_″ confirms that the charge accumulation and heterogeneous interface of RGO and Fe generates space charge polarization loss. Based on the Debye relaxation theory, the existence of polarization can be proved by the Cole–Cole semicircle, each semicircle of Cole–Cole expresses a Debye relaxation process [60]. Figure S12 and Table S2 show the *ε*′–*ε*″ curves of RGO, Fe/RGO-1, Fe/RGO-2, Fe/RGO-3 and Fe/RGO-4, it can be seen that the number of semicircles increases with the increase in Fe, indicating an enhanced relaxation process compared with RGO. And the conductive loss increases with the increase in conductivity and the polarization loss intensity decreases (the *ε*′–*ε*″ curve becomes smooth). From the first-principle calculation and carrier transfer characterization, it can be seen that the interfacial electron aggregation and electric field-derived flowing dominated the polarization loss by the carrier injection mechanism of Fe/graphene. We focused on exploring the Fe/RGO interface structure and plotted its electric field and charge density distributions using electron holography (Fig. 6d–i). It is obviously observed that there are positive and negative charge distribution around the interfacial region of carbon layer and Fe nanosheet forming an out-plane electric field within the carrier collector interface, leading to an enhanced interfacial polarization relaxation. Unbalanced charge distribution due to electron migration can accumulate around the heterogeneous interface. Under the EM field, the electric dipole turns repeatedly to promote the interfacial polarization so as to considerably consume the EM wave energy. More importantly, increasing attenuation capacity and improving impedance matching characteristics are key factors to achieve excellent EMW absorption performance. The tan *δ*_*ε*_ and *α* values of the Fe/RGO composites increase with the increase in Fe content (Fig. S13). The partial electrons of Fe injected into the graphene vibrate and produce polarization relaxation under microwave, significantly affecting the interface polarization loss and conductance loss. However, more interfaces can obtain high dielectric loss, but the absorption performance is not further improved mainly due to impedance mismatch (the low *Z* values), which is consistent with our previous work [61]. Therefore, Fe/RGO-2 and Fe/RGO-3 have excellent absorbing performance due to its suitable attenuation characteristics and impedance matching. For comparison, the absorption properties of Fe/RGO′ composites without heterogeneous interfacial interaction were investigated by mechanical mixing in the same ratio as above with 2 wt% filler loading. The *ε*′ and *ε*″ curves of Fe/RGO-1′, Fe/RGO-2′, Fe/RGO-3′, and Fe/RGO-4′ change weakly and does not exhibit dielectric dispersion behavior with the increase in Fe content (Fig. S14). Therefore, there exists an interfacial electronic interaction between RGO and Fe in Fe/RGO composites, which would affect the dielectric properties of RGO and thus have excellent absorption properties.

**Fig. 7:**
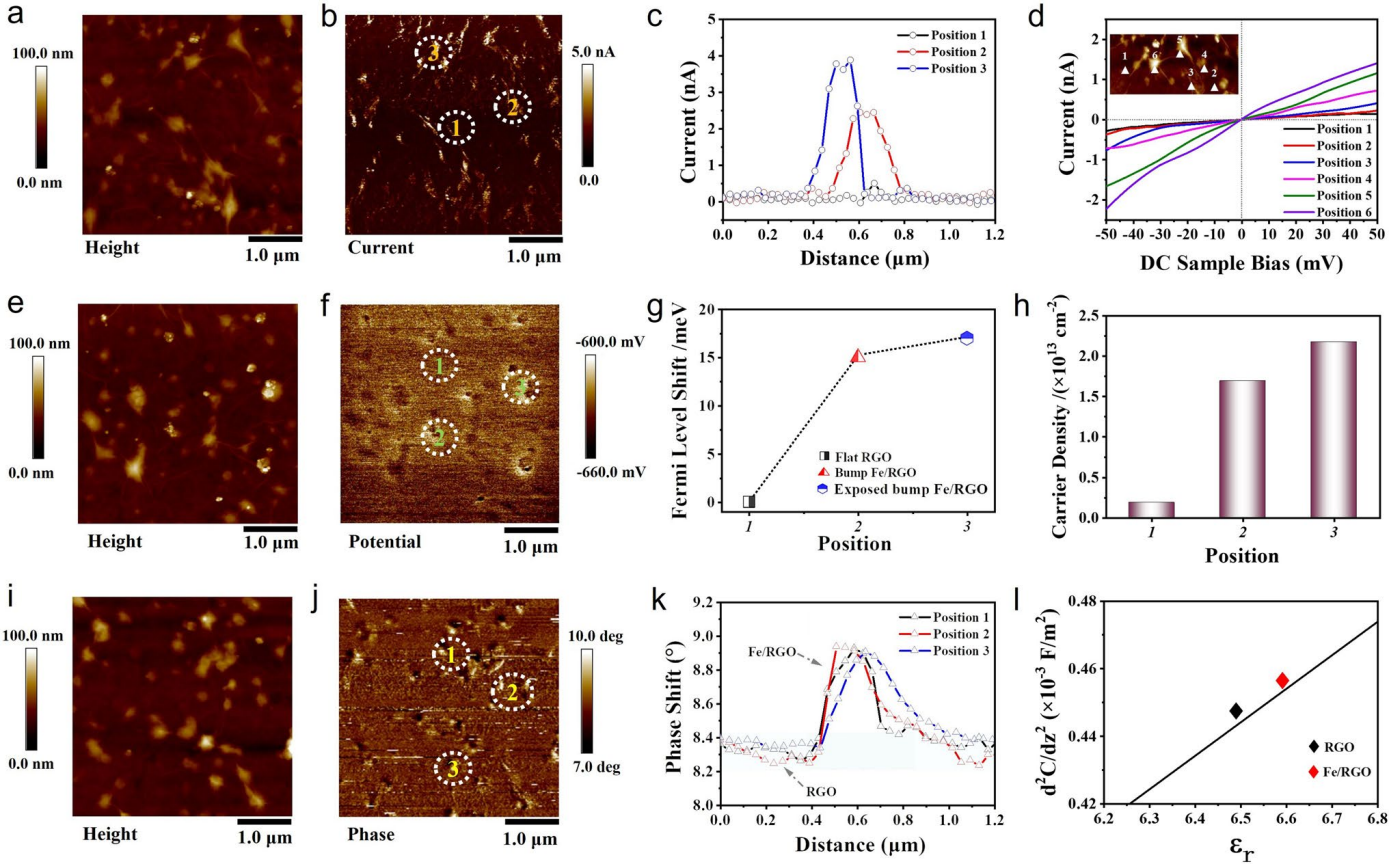
Electrical measurement of the Fe/RGO. **a**, **e**, **i** AFM topographic images of Fe/RGO. **b** C-AFM current distribution image. The current distribution curves **c** and *I*–*V* curves **d** of the calibration position region. **f** KPFM surface potential image. **g** The shift of the RGO Fermi level and **h** carrier density values varies with position. **j** and **k** ${\Delta }\phi$ distribution images and curves of calibration position region of Fe/RGO, **l** dielectric response curve of EFM

Based on the above mechanism analysis, in order to further clarify the mechanism of graphene’s electronic structure and dielectric property response behavior, this work explored the electrical transport properties of Fe/RGO at micro–nanoscale. Electrostatic interactions have been widely used to characterize the conductivity, potential distribution, surface charge distribution and dielectric properties of various samples by C-AFM, KPFM and EFM measurements, etc. The surface topography and the corresponding current distribution images of Fe/RGO can be obtained by scanning the micro-area surface with C-AFM, and the *I*–*V* curves can be further obtained at specific points. As shown in Fig. 7a–d, the conductivity has a significant difference in different regions as marked by the white lines. The current is lower in the RGO area (dark area) and higher in the close contact area of the Fe/RGO interface (light area). In addition, the *I*–*V* curves of Fe/RGO surface region show that the current of Fe/RGO increases with the increase in contact area and binding tightness, indicating that the electron migration and hopping jointly determine the conductivity of RGO, which is consistent with the electrical properties analysis results of first principles calculation, FG-FET and powders conductivity analysis (Fig. S15). It can be seen from the topographical height and surface potential distribution images of Fe/RGO that RGO layers show uniform surface potential at the same location, which indicates that the electronic properties of RGO is not significantly affected by air doped charges or substrate defects (Fig. 7e, f). The surface potential of Fe/RGO region increases with the position bulge of Fe, indicating that the degree of charge transfer between Fe and RGO increases due to the interface continuity. Figure 7g shows the relative Fermi level offset of Fe/RGO with the interface contact position according to the Fermi level formula [62]: $n\left( {E_{{\text{F}}} } \right) = \frac{1}{\pi }\left( {\frac{{E_{{\text{F}}} }}{{\hbar V_{{\text{F}}} }}} \right)^{2}$, where $V_{{\text{F}}}$ and $\hbar$ represent Fermi velocity and Planck constant. It can be calculated that the carrier densities of Fe/RGO in different regions are about 0.18 × 10^13^, 1.69 × 10^13^ and 2.18 × 10^13^ cm^−2^, respectively (Fig. 7h). The surface charge distribution and dielectric properties of Fe/RGO in different regions are investigated under the influence of conductive probe by EFM. The measured phase change is defined as the difference between the actual and the free vibration phase of the probe. The influence of the interaction between the probe and the sample surface on the probe motion is explained by introducing a force constant, and the formula is as follows [63]:

7 $$\Delta \phi \approx \frac{Q}{k} \cdot \frac{{2k^{\prime}q_{{{\text{tip}}}} q_{{{\text{surf}}}} }}{{z^{3} }} \propto q_{{{\text{surf}}}}$$

where ${\Delta }\phi$ represents the phase difference, *Q* and *k* are the quality factors and force constants of the probe, $k^{\prime}$ is the electrostatic force constant, and $q_{{{\text{tip}}}}$ and $q_{{{\text{surf}}}}$ represent the surface charges of the probe and sample. It can be seen from the above equation that the phase difference increases with the increase in the surface charge density of the sample. Therefore, it can be seen from Fig. 7i–k that the charge is mostly distributed in the convex part of RGO coated Fe surface, while the charge is less distributed in the flat part of RGO, indicating that the presence of Fe increases the conductivity of RGO. Considering only electrostatic force effect, the capacitance model can be considered between the probe and the sample, and the relationship between the phase difference (${\Delta }\phi \left( {2{\upomega }} \right)$) and permittivity ($\varepsilon_{r}$) can be expressed as [64]:

8 $$\Delta \phi \left( {2\omega } \right) = \frac{Q}{4k}\frac{{{\text{d}}^{2} C}}{{{\text{d}}z^{2} }}V^{2}_{{{\text{AC}}}} \cos \left( {2\omega t} \right)$$

9 $$C = 2\pi \varepsilon_{0} R\ln \left[ {1 + \frac{{R\left( {1 - \sin \theta_{0} } \right)}}{{z + h/\varepsilon_{r} }}} \right]$$

10 $$\frac{{{\text{d}}^{2} C}}{{{\text{d}}z^{2} }} = 2\pi \varepsilon_{0} R^{2} \left( {1 - \sin \theta_{0} } \right)\frac{{2\left( {z + h/\varepsilon_{r} } \right) + R\left( {1 - \sin \theta_{0} } \right)}}{{\left[ {\left( {z + h/\varepsilon_{r} } \right)^{2} + \left( {z + h/\varepsilon_{r} } \right)R\left( {1 - \sin \theta_{0} } \right)} \right]^{2} }}$$

where $\theta_{0}$ and $R$ are the conical tip angle and tip radius of the probe. $\varepsilon_{0}$ is the permittivity of the vacuum, and $C$ is the capacitance between substrate and probe. $h$ and $\varepsilon_{r}$ are the local thickness and relative permittivity, and $V_{{{\text{AC}}}}$ is constant during scanning. The parameters of the local dielectric detection are shown in Table S1. From Fig. 7l, the average permittivity of RGO and Fe/RGO in different regions can be roughly calculated according to the $\frac{{{\text{d}}^{2} C}}{{{\text{dz}}^{2} }} - \varepsilon_{r}$ fitting formula, and the calculated results are 6.49 and 6.58. The permittivity of Fe/RGO region is higher than that of RGO, which is consistent with the changing trend of dielectric parameters in Figs. 3 and 5. Therefore, it can be inferred that Fe/RGO heterogeneous interfaces with work function differences and orbital hybrid interactions promote carrier injection and redistribution in the form of ohmic contact, which enhances conductance loss, interface and dipole polarization loss and provides infinite possibilities for the tunability of dielectric and absorption properties. At the same time, we also explored the influence of trace transition metal elements (Co and Ni) on the dielectric properties of graphene, as shown in Fig. S16. The results showed that the dielectric dispersion characteristic of graphene was improved greatly by the carrier injection mechanism resulting in significant absorption performance, which indicates that the mechanism is of universal adaptability to the improvement of graphene dielectric properties.

**Fig. 8:**
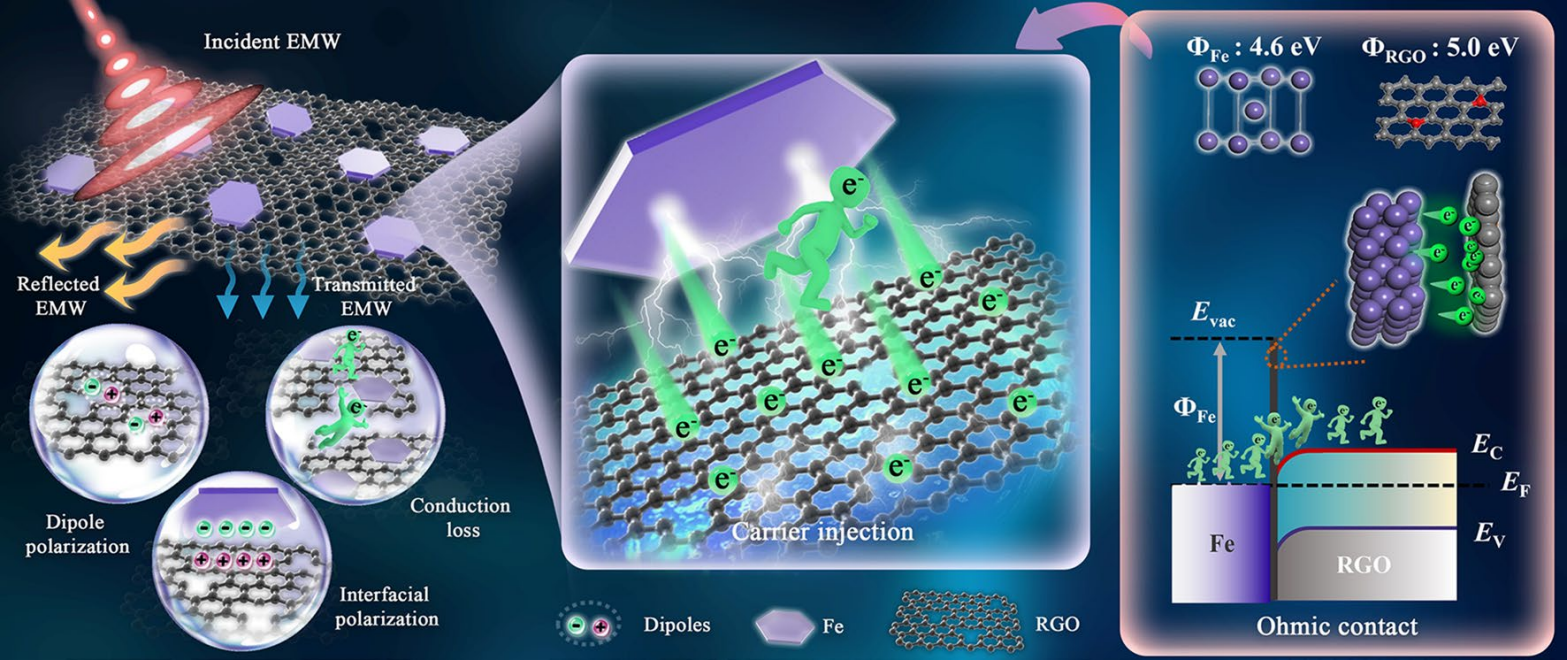
Schematic diagram of EMW absorption of Fe/RGO

To have a better understanding of EMW absorption mechanism for Fe/RGO, a schematic diagram exhibiting is shown in Fig. 8. The excellent absorption properties of Fe/RGO composites can be attributed to conduction loss, polarization relaxation and better impedance matching. Firstly, the polarization relaxation related to the heterogeneous interfaces formed between RGO and Fe is beneficial in enhancing the interface polarization. Secondly, the improved conductivity caused by the carrier injection mechanism (including electron migration and hopping) effectively enhances the conductance loss, and the resulting charge aggregation and rearrangement further increases the interface polarization loss. This phenomenon is mainly attributed to the ohmic contact form between the RGO and Fe interface based on the difference of work function, which allows the free electrons in Fe to be better injected into the graphene. Thirdly, the unique lamellar structure of RGO can effectively improve the electron transport efficiency, resulting in high conductive loss. Fourthly, the dipole polarization caused by defects in the edge and in-plane regions of Fe/RGO increases the dielectric loss. Furthermore, the proper complex permittivity of Fe/RGO adjusted by Fe content provided an optimal impedance matching, which helps incident microwaves to enter the Fe/RGO composite. Consequently, multiple loss mechanisms and good impedance matching in Fe/RGO can be obtained significant EMW absorption performance. Figure S17 summarizes the EMW absorption properties of RGO-based composites reported in the recent literature (Table S3). Compared with other RGO absorbers, the Fe/RGO composite in this study has lightweight and broadband microwave absorption properties at a low fill loading (2 wt%), proving the feasibility of our strategy.

## Conclusion

In summary, this work designed and developed a carrier injection strategy for fully exploiting the dielectric properties of graphene based on the theoretical guidance of the different electron gain and loss capabilities. By regulating the amount of Fe nanosheets in Fe/RGO, the optimizing dielectric dispersion properties of graphene are achieved through carrier injection mechanism formed by ohmic contact between RGO and Fe interface. It is revealed that electron migration directly increases the conductivity of graphene at the Fe/RGO interface and the resulting charge accumulation and rearrangement effectively increase the conductivity loss, dipole polarization and interfacial polarization loss, and the electron holography confirmed that the Fe/RGO possessed significant charge distribution around the interface location compared to RGO. As a result, the Fe/RGO-2 exhibits excellent EMW absorption performance at a low loading of 2 wt% that the RL_min_ value can reach − 53.38 dB at 13.56 GHz (2.45 mm) and the EAB is 7.52 GHz (10.48–18 GHz, covering the full *Ku* band) at 2.62 mm. This present work elucidates an intrinsic insight into the dielectric polarization behavior of graphene-based materials and provides a reliable strategy for the controllable fabrication of novel lightweight EMW absorbing materials.

## Supplementary Information

Below is the link to the electronic supplementary material.

Supplementary file1 (PDF 2074 KB)

## References

[1] X. Yang, Y. Duan, S. Li, H. Pang, L. Huang et al., Bio-inspired microwave modulator for high-temperature electromagnetic protection, infrared stealth and operating temperature monitoring. Nano-Micro Lett. **14**(1), 28 (2021). 10.1007/s40820-021-00776-3 10.1007/s40820-021-00776-3 PMC8669058 34902068

[2] Y. Liu, X. Huang, X. Yan, L. Xia, T. Zhang et al., Pushing the limits of microwave absorption capability of carbon fiber in fabric design based on genetic algorithm. J. Adv. Ceram. **12**(2), 329–340 (2023). 10.26599/jac.2023.9220686

[3] P. Song, Z. Ma, H. Qiu, Y. Ru, J. Gu, High-efficiency electromagnetic interference shielding of rGO@FeNi/epoxy composites with regular honeycomb structures. Nano-Micro Lett. **14**(1), 51 (2022). 10.1007/s40820-022-00798-5 10.1007/s40820-022-00798-5 PMC8795265 35084576

[4] J. Cheng, H. Zhang, H. Wang, Z. Huang, H. Raza et al., Tailoring self-polarization of bimetallic organic frameworks with multiple polar units toward high-performance consecutive multi-band electromagnetic wave absorption at gigahertz. Adv. Funct. Mater. **32**(24), 2201129 (2022). 10.1002/adfm.202201129

[5] C. Hou, J. Cheng, H. Zhang, Z. Lu, X. Yang et al., Biomass-derived carbon-coated WS_2_ core-shell nanostructures with excellent electromagnetic absorption in C-band. Appl. Surf. Sci. **577**, 151939 (2022). 10.1016/j.apsusc.2021.151939

[6] D. Zhang, T. Liu, M. Zhang, H. Zhang, X. Yang et al., Confinedly growing and tailoring of Co_3_O_4_ clusters-WS_2_ nanosheets for highly efficient microwave absorption. Nanotechnology **31**(32), 325703 (2020). 10.1088/1361-6528/ab8b8d 32315995 10.1088/1361-6528/ab8b8d

[7] Z. Huang, J. Cheng, H. Zhang, Y. Xiong, Z. Zhou et al., High-performance microwave absorption enabled by Co_3_O_4_ modified VB-group laminated VS_2_ with frequency modulation from S-band to Ku-band. J. Mater. Sci. Technol. **107**, 155–164 (2022). 10.1016/j.jmst.2021.08.005

[8] H. Zhang, T. Liu, Z. Huang, J. Cheng, H. Wang et al., Engineering flexible and green electromagnetic interference shielding materials with high performance through modulating WS_2_ nanosheets on carbon fibers. J. Materiomics **8**(2), 327–334 (2022). 10.1016/j.jmat.2021.09.003

[9] G. Bellis, A. Tamburrano, A. Dinescu, M.L. Santarelli, M.S. Sarto, Electromagnetic properties of composites containing graphite nanoplatelets at radio frequency. Carbon **49**(13), 4291–4300 (2011). 10.1016/j.carbon.2011.06.008

[10] F. Ye, Q. Song, Z. Zhang, W. Li, S. Zhang et al., Direct growth of edge-rich graphene with tunable dielectric properties in porous Si_3_N_4_ ceramic for broadband high-performance microwave absorption. Adv. Funct. Mater. **28**, 1707205 (2018). 10.1002/adfm.201707205

[11] Y. Dai, M. Sun, C. Liu, Z. Li, Electromagnetic wave absorbing characteristics of carbon black cement-based composites. Cem. Concr. Compos. **32**(7), 508–513 (2010). 10.1016/j.cemconcomp.2010.03.009

[12] T. Zhao, C. Hou, H. Zhang, R. Zhu, S. She et al., Electromagnetic wave absorbing properties of amorphous carbon nanotubes. Sci. Rep. **4**, 5619 (2014). 10.1038/srep05619 25007783 10.1038/srep05619 PMC4090627

[13] A.K. Geim, K.S. Novoselov, The rise of graphene. Nat. Mater. **6**(3), 183–191 (2007). 10.1038/nmat1849 17330084 10.1038/nmat1849

[14] C.G. Lee, X.D. Wei, J.W. Kysar, J. Hone, Measurement of the elastic properties and intrinsic strength of monolayer graphene. Science **321**(5887), 385–388 (2008). 10.1126/science.1157996 18635798 10.1126/science.1157996

[15] A.K. Geim, Graphene: status and prospects. Science **324**(5934), 1530–1534 (2009). 10.1126/science.1158877 19541989 10.1126/science.1158877

[16] R. Murali, Y. Yang, K. Brenner, T. Beck, J.D. Meindl, Breakdown current density of graphene nanoribbons. Appl. Phys. Lett. **94**, 243114 (2009). 10.1063/1.3147183

[17] Q. Li, Y. Zhao, X. Li, L. Wang, X. Li et al., MOF induces 2D GO to assemble into 3D accordion-like composites for tunable and optimized microwave absorption performance. Small **16**(42), e2003905 (2020). 10.1002/smll.202003905 32996264 10.1002/smll.202003905

[18] X. Liu, Y. Huang, L. Ding, X. Zhao, P. Liu et al., Synthesis of covalently bonded reduced graphene oxide-Fe_3_O_4_ nanocomposites for efficient electromagnetic wave absorption. J. Mater. Sci. Technol. **72**, 93–103 (2021). 10.1016/j.jmst.2020.09.012

[19] Y. Ge, H. Wang, T. Wu, B. Hu, Y. Shao et al., Accordion-like reduced graphene oxide embedded with Fe nanoparticles between layers for tunable and broadband electromagnetic wave absorption. J. Colloid Interface Sci. **628**, 1019–1030 (2022). 10.1016/j.jcis.2022.08.020 36049278 10.1016/j.jcis.2022.08.020

[20] D. Xu, S. Yang, P. Chen, Q. Yu, X. Xiong et al., Synthesis of magnetic graphene aerogels for microwave absorption by in-situ pyrolysis. Carbon **146**, 301–312 (2019). 10.1016/j.carbon.2019.02.005

[21] G. Pan, J. Zhu, S. Ma, G. Sun, X. Yang, Enhancing the electromagnetic performance of Co through the phase-controlled synthesis of hexagonal and Cubic Co nanocrystals grown on graphene. ACS Appl. Mater. Interfaces **5**(23), 12716–12724 (2013). 10.1021/am404117v 24266516 10.1021/am404117v

[22] I. Arief, S. Biswas, S. Bose, FeCo-anchored reduced graphene oxide framework-based soft composites containing carbon nanotubes as highly efficient microwave absorbers with excellent heat dissipation ability. ACS Appl. Mater. Interfaces **9**(22), 19202–19214 (2017). 10.1021/acsami.7b04053 28520409 10.1021/acsami.7b04053

[23] B. Wen, M. Cao, M. Lu, W. Cao, H. Sh et al., Reduced graphene oxides: Light-weight and high-efficiency electromagnetic interference shielding at elevated temperatures. Adv. Mater. **26**, 3484–3489 (2014). 10.1002/adma.201400108 24648151 10.1002/adma.201400108

[24] B. Wen, M. Cao, Z. Hou, W. Song, L. Zhang et al., Temperature dependent microwave attenuation behavior for carbon-nanotube/silica composites. Carbon **65**, 124–139 (2013). 10.1016/j.carbon.2013.07.110

[25] M. Cao, W. Song, Z. Hou, B. Wen, J. Yuan, The effects of temperature and frequency on the dielectric properties, electromagnetic interference shielding and microwave-absorption of short carbon fiber/silica composites. Carbon **48**(3), 788–796 (2010). 10.1016/j.carbon.2009.10.028

[26] X. Zhao, Z. Zhang, L. Wang, K. Xi, Q. Cao et al., Excellent microwave absorption property of graphene-coated Fe nanocomposites. Sci. Rep. **3**, 3421 (2013). 10.1038/srep03421 24305606 10.1038/srep03421 PMC3852363

[27] G. Ni, Y. Zheng, S. Bae, C. Tan, O. Kahya et al., Graphene-ferroelectric hybrid structure for flexible transparent electrodes. ACS Nano **6**(5), 3935–3942 (2012). 10.1021/nn3010137 22524641 10.1021/nn3010137

[28] H. Kim, H.H. Kim, J.I. Jang, S.K. Lee, G.W. Lee et al., Doping graphene with an atomically thin two dimensional molecular layer. Adv. Mater. **26**, 8141–8146 (2014). 10.1002/adma.201403196 25243356 10.1002/adma.201403196

[29] M. Kim, K.J. Kim, S.J. Lee, H.M. Kim, S.Y. Cho et al., Highly stable and effective doping of graphene by selective atomic layer deposition of ruthenium. ACS Appl. Mater. Interfaces **9**(1), 701–709 (2017). 10.1021/acsami.6b12622 27936584 10.1021/acsami.6b12622

[30] Y. Ren, S. Chen, W. Cai, Y. Zhu, C. Zhu et al., Controlling the electrical transport properties of graphene by in situ metal deposition. App. Phys. Lett. (2010). 10.1063/1.3471396

[31] Q. Li, J. Tan, Z. Wu, L. Wang, W. You et al., Hierarchical magnetic-dielectric synergistic Co/CoO/RGO microspheres with excellent microwave absorption performance covering the whole X band. Carbon **201**, 150–160 (2023). 10.1016/j.carbon.2022.08.090

[32] Z. Xiang, J. Xiong, B. Deng, E. Cui, L. Yu et al., Rational design of 2D hierarchically laminated Fe_3_O_4_@nanoporous carbon@rGO nanocomposites with strong magnetic coupling for excellent electromagnetic absorption applications. J. Mater. Chem. C **8**(6), 2123–2134 (2020). 10.1039/c9tc06526a

[33] P. Liu, S. Gao, G. Zhang, Y. Huang, W. You et al., Hollow engineering to Co@N-doped carbon nanocages via synergistic protecting-etching strategy for ultrahigh microwave absorption. Adv. Funct. Mater. **31**, 2102812 (2021). 10.1002/adfm.202102812

[34] L. Wang, X. Yu, M. Huang, W. You, Q. Zeng et al., Orientation growth modulated magnetic-carbon microspheres toward broadband electromagnetic wave absorption. Carbon **172**, 516–528 (2021). 10.1016/j.carbon.2020.09.050

[35] R. Che, L. Peng, X. Duan, Q. Chen, X. Liang, Microwave absorption enhancement and complex permittivity and permeability of Fe encapsulated within carbon nanotubes. Adv. Mater. **16**(5), 401–405 (2004). 10.1002/adma.200306460

[36] W. Li, H. Qi, F. Guo, Y. Du, N. Song et al., Co nanoparticles supported on cotton-based carbon fibers: a novel broadband microwave absorbent. J. Alloys Compd. **772**, 760–769 (2019). 10.1016/j.jallcom.2018.09.075 10.1039/c9ra05844c PMC9072117 35531530

[37] S. Qiu, H. Lyu, J. Liu, Y. Liu, N. Wu et al., Facile synthesis of porous Nickel/Carbon composite microspheres with enhanced electromagnetic wave absorption by magnetic and dielectric losses. ACS Appl. Mater. Interfaces **8**(31), 20258–20266 (2016). 10.1021/acsami.6b03159 27441382 10.1021/acsami.6b03159

[38] F. Wang, N. Wang, X. Han, D. Liu, Y. Wang et al., Core-shell FeCo@carbon nanoparticles encapsulated in polydopamine-derived carbon nanocages for efficient microwave absorption. Carbon **145**, 701–711 (2019). 10.1016/j.carbon.2019.01.082

[39] H. Lv, X. Liang, Y. Cheng, H. Zhang, D. Tang et al., Coin-like α-Fe_2_O_3_@CoFe_2_O_4_ core–shell composites with excellent electromagnetic absorption performance. ACS Appl. Mater. Interfaces **7**(8), 4744–4750 (2015). 10.1021/am508438s 25664491 10.1021/am508438s

[40] K. Pi, K.M. McCreary, W. Bao, W. Han, Y.F. Chiang et al., Electronic doping and scattering by transition metals on graphene. Phys. Rev. B **80**, 075406 (2009). 10.1103/PhysRevB.80.075406

[41] K. Zhang, W. Lv, J. Chen, H. Ge, C. Chu et al., Synthesis of RGO/AC/Fe_3_O_4_ composite having 3D hierarchically porous morphology for high effective electromagnetic wave absorption. Compos. Part B-Eng. **169**, 1–8 (2019). 10.1016/j.compositesb.2019.03.081

[42] C. Wang, W. Chen, C. Han, G. Wang, B. Tang et al., Growth of millimeter-size single crystal graphene on cu foils by circumfluence chemical vapor deposition. Sci. Rep. **4**, 4537 (2014). 10.1038/srep04537 24686949 10.1038/srep04537 PMC3971397

[43] J.W. Suk, A. Kitt, C.W. Magnuson, Y. Hao, S. Ahmed et al., Transfer of CVD-grown monolayer graphene onto arbitrary substrates. ACS Nano **5**(9), 6916–6924 (2011). 10.1021/nn201207c 21894965 10.1021/nn201207c

[44] C. Wang, Y. Liu, Z. Jia, W. Zhao, G. Wu, Multicomponent nanoparticles synergistic one-dimensional nanofibers as heterostructure absorbers for tunable and efficient microwave absorption. Nano-Micro Lett. **15**(1), 13 (2022). 10.1007/s40820-022-00986-3 10.1007/s40820-022-00986-3 PMC9755410 36520259

[45] G. Qin, X. Huang, X. Yan, Y. He, Y. Liu et al., Carbonized wood with ordered channels decorated by NiCo_2_O_4_ for lightweight and high-performance microwave absorber. J. Adv. Ceram **11**(1), 105–119 (2021). 10.1007/s40145-021-0520-z

[46] T. Gao, R. Zhao, Y. Li, Z. Zhu, C. Hu et al., Sub-nanometer Fe clusters confined in carbon nanocages for boosting dielectric polarization and broadband electromagnetic wave absorption. Adv. Funct. Mater. **32**, 22043 (2022). 10.1002/adfm.202204370

[47] Z. Yan, Z. Xu, Z. Yang, L. Yue, L. Huang, Graphene oxide/Fe_2_O_3_ nanoplates supported Pt for enhanced room-temperature oxidation of formaldehyde. Appl. Surf. Sci. **467–468**, 277–285 (2019). 10.1016/j.apsusc.2018.10.123

[48] W. Xue, H. He, J. Zhu, P. Yuan, FTIR investigation of CTAB-Al-montmorillonite complexes. Spectrochim. Acta A Mol. Biomol. Spectrosc. **67**, 1030–1036 (2007). 10.1016/j.saa.2006.09.024 17289428 10.1016/j.saa.2006.09.024

[49] H. Quan, B. Cheng, Y. Xiao, S. Lei, One-pot synthesis of α-Fe_2_O_3_ nanoplates-reduced graphene oxide composites for supercapacitor application. Chem. Eng. J. **286**, 165–173 (2016). 10.1016/j.cej.2015.10.068

[50] L. Lei, Z. Yao, J. Zhou, W. Zheng, B. We et al., Hydrangea-like Ni/Nio/C composites derived from metal–organic frameworks with superior microwave absorption. Carbon **173**, 69–79 (2021). 10.1016/j.carbon.2020.10.093

[51] B. Du, M. Cai, X. Wang, J. Qian, C. He, A. Shui, Enhanced electromagnetic wave absorption property of binary ZnO/NiCo_2_O_4_ composites. J. Adv. Ceram. **10**(4), 832–842 (2021). 10.1007/s40145-021-0476-z

[52] H. Xu, G. Zhang, Y. Wang, M. Ning, B. Ouyang et al., Size-dependent oxidation-induced phase engineering for MOFs derivatives via spatial confinement strategy toward enhanced microwave absorption. Nano-Micro Lett. **14**(1), 102 (2022). 10.1007/s40820-022-00841-5 10.1007/s40820-022-00841-5 PMC9005575 35412156

[53] J. Cheng, C. Li, Y. Xiong, H. Zhang, H. Raza et al., Recent advances in design strategies and multifunctionality of flexible electromagnetic interference shielding materials. Nano-Micro Lett. **14**(1), 1–31 (2022). 10.1007/s40820-022-00823-7 10.1007/s40820-022-00823-7 PMC8956783 35333993

[54] D. Zhang, T. Liu, J. Cheng, J. Chai, X. Yang et al., Light-weight and low-cost electromagnetic wave absorbers with high performances based on biomass-derived reduced graphene oxides. Nanotechnology **30**(44), 445708 (2019). 10.1088/1361-6528/ab35fa 31349245 10.1088/1361-6528/ab35fa

[55] D. Zhang, H. Wang, J. Cheng, C. Han, X. Yang et al., Conductive WS_2_-NS/CNTs hybrids based 3D ultra-thin mesh electromagnetic wave absorbers with excellent absorption performance. Appl. Surf. Sci. **528**, 147052 (2020). 10.1016/j.apsusc.2020.147052

[56] J.S. Galsin, Free-electron theory of metals, in *Solid State Physics*. ed. by J.S. Galsin (Elsevier, Amsterdam, 2019), pp.177–198. 10.1016/B978-0-12-817103-5.00009-8

[57] H. Lv, Z. Yang, S.J.H. Ong, C. Wei, H. Liao et al., A flexible microwave shield with tunable frequency-transmission and electromagnetic compatibility. Adv. Funct. Mater. **29**, 1900163 (2019). 10.1002/adfm.201900163

[58] J. Cheng, H. Zhang, M. Ning, H. Raza, D. Zhang et al., Emerging materials and designs for low- and multi-band electromagnetic wave absorbers: The search for dielectric and magnetic synergy? Adv. Funct. Mater. **32**(23), 2200123 (2022). 10.1002/adfm.202200123

[59] Y. Wu, Y. Zhao, M. Zhou, S. Tan, R. Peymanfar et al., Ultrabroad microwave absorption ability and infrared stealth property of nano-micro Cus@rGO lightweight aerogels. Nano-Micro Lett. **14**(1), 171 (2022). 10.1007/s40820-022-00906-5 10.1007/s40820-022-00906-5 PMC9392679 35987861

[60] T. Xu, J. Li, D. Zhao, X. Chen, G. Sun et al., Structural engineering enabled bimetallic (Ti_1-γ_Nb_γ_)_2_AlC solid solution structure for efficient electromagnetic wave absorption in gigahertz. Small **19**, e2300119 (2023). 10.1002/smll.202300119 36974601 10.1002/smll.202300119

[61] X. Yan, X. Huang, Y. Chen, Y. Liu, L. Xia et al., A theoretical strategy of pure carbon materials for lightweight and excellent absorption performance. Carbon **174**, 662–672 (2021). 10.1016/j.carbon.2020.11.044

[62] X. Qiu, G. Qi, Y. Yang, C. Wang, Electrostatic characteristics of nanostructures investigated using electric force microscopy. J. Solid State Chem. **181**(7), 1670–1677 (2008). 10.1016/j.jssc.2008.06.036

[63] S.N. Magonov, V. Elings, M.H. Whangbo, Phase imaging and stiffness in tapping-mode atomic force microscopy. Surf. Sci. **375**, L385–L391 (1997). 10.1016/S0039-6028(96)01591-9

[64] S. Peng, Q. Zeng, X. Yang, J. Hu, X. Qiu et al., Local dielectric property detection of the interface between nanoparticle and polymer in nanocomposite dielectrics. Sci. Rep. **6**, 38978 (2016). 10.1038/srep38978 27958347 10.1038/srep38978 PMC5154196

