# Supplementary material for: Tracking Regulatory Mechanism of Trace Fe on Graphene Electromagnetic Wave Absorption
Source: Nanomicro Lett. 2024 Jan 4;16:66. doi: 10.1007/s40820-023-01280-6 (PMC10767016; doi:10.1007/s40820-023-01280-6)
Supplement: Supplementary file 1 — Supplementary file1 (PDF 2074 KB) [file 40820_2023_1280_MOESM1_ESM.docx]

Supporting Information for

**Tracking Regulatory Mechanism of Trace Fe on Graphene Electromagnetic Wave Absorption**

Kaili Zhang^1, 2^, Yuhao Liu^1, 2^, Yanan Liu^1, 2^, Yuefeng Yan^1, 2^, Guansheng Ma^1, 2^, Bo Zhong^3^, Renchao Che^4,^ *, Xiaoxiao Huang^1, 2,^ *

^1^ School of Materials Science and Engineering, Harbin Institute of Technology, Harbin, 150001, PR China

^2^ MIIT Key Laboratory of Advanced Structural-Functional Integration Materials & Green Manufacturing Technology, Harbin Institute of Technology, Harbin, 150001, PR China

^3^ School of Materials Science and Engineering, Harbin Institute of Technology at Weihai, Weihai, 264209, PR China

^4^ Laboratory of Advanced Materials, Shanghai Key Lab of Molecular Catalysis and Innovative Materials, Fudan University, Shanghai, 200438, PR China

*Corresponding authors.

E-mail: swliza@hit.edu.cn (Xiaoxiao Huang);

E-mail: rcche@fudan.edu.cn (Renchao Che)

**S1 Experimental Section**

**S1.1 Preparation of Co_2_O_3_ nanosheets and the surface modification by CTAB**

1.9 g CoCl_2_·6H_2_O and 0.8 g NaOH were added into the 160 mL deionized water and stirred till solid particle were completely dissolved. Next, the mixture was transferred to Teflon-lined stainless-steel autoclave for solvothermal reaction at 120 °C for 3 h. The autoclave was cooled to ambient temperature naturally. The product was successively washed by ethanol and distilled water for 3 times and then dried at 60°C. Co_2_O_3_ nanosheets were obtained when the as-synthesized Co(OH)_2_ nanosheets were put into the tube furnace and heat treated at 290 ℃ for 2h under air atmosphere. For the surface modification, Co_2_O_3_ nanosheets (1.0 g) and hexadecyltrimethylammonium bromide (CTAB, 0.5 g) were dispersed in deionized water (100 mL) with continuous magnetic stirring in a 60 °C water bath for 2 h. The product was washed with deionized water for several times and dried at 65 °C, and finally the modified hexagonal Co_2_O_3_ nanosheets were obtained.

**S1.2 Preparation of Ni_2_O_3_ nanosheets and the surface modification by CTAB**

2.0 g Ni(NO_3_)_2_·6H₂O was added into the 100 mL deionized water and stirred till solid particle were completely dissolved. With continuous magnetic stirring, 5 mL NH_3_·H_2_O was dropwise added to the solution. The mixture was transferred to Teflon-lined stainless-steel autoclave for solvothermal reaction at 150 °C for 12 h. The autoclave was cooled to ambient temperature naturally. The product was successively washed by ethanol and distilled water for 3 times and then dried at 60°C. Ni_2_O_3_ nanosheets were obtained when the as-synthesized Ni(OH)_2_ nanosheets were put into the tube furnace and heat treated at 350℃ for 2h under air atmosphere. For the surface modification, Ni_2_O_3_ nanosheets (1.0 g) and hexadecyltrimethylammonium bromide (CTAB, 0.5 g) were dispersed in deionized water (100 mL) with continuous magnetic stirring in a 60 °C water bath for 2 h. The product was washed with deionized water for several times and dried at 65 °C, and finally the modified hexagonal Ni_2_O_3_ nanosheets were obtained.

**S1.3 Preparation of Co/RGO and Ni/RGO composite**

GO suspension (100 mL, 10 mg·mL^-1^) were mixed with modified Co_2_O_3_ (4 mg) and Ni_2_O_3_ (4 mg) respectively, and the mixture was ultrasonic stirred evenly for 3 h by electrostatic adsorption process. Then the mixtures were sealed and refrigerated in the refrigerator, and treated by freeze-drying method (-65 ℃, 0.1 Pa) for 24 h. The above products were put into the tube furnace and heat treated at 350 ℃ for 2h under H_2_ atmosphere (60 mL·min^-1^). Finally, Co/RGO and Ni/RGO were obtained, respectively.

**S1.4 Calculation details**

All of the calculation results of Co/graphene and Ni/graphene were carried out by the Castep module of Materials Studio software based on density functional theory (DFT). The ion−electron interactions were described by the OTFG ultrasoft pseudopotential. The Generalized Gradient Approximation (GGA) method with Perdew−Burke−Ernzerhof (PBE) functional was adopted to solve the exchange and correlation functional energies. The cutoff energy and k-point mesh were determined as 500 eV and 2×3×1, respectively. The calculation accuracy was 1.0×10^−5^ eV per atom. The thickness of the vacuum layer along the c-axis is fixed at 20 Å.

**S2 Supplementary Figures and Tables**

**Fig. S1.** SEM images of **a** RGO, **b** Fe/RGO-1, **c** Fe/RGO-2, **d** Fe/RGO-3 and **e** Fe/RGO-4. **f** ICP measurement of samples.

**Fig. S2.** XRD patterns **a, b** and Raman spectra **c, d** of samples.

**Fig. S3.** **a** XPS survey, **b** C 1s spectra and **c** O 1s spectra of GO.

**Fig. S4.** Real part (µ') and imaginary part (µ") of permeability and permeability loss parameter (tan δ_μ_) of **a** RGO composites and **b** Fe/RGO-2 composites at a low filling loading of 1-5 wt%.

**Fig. S5.** RL value versus frequency for specific thickness values of **a** RGO composites and **b** Fe/RGO-2 composites at a low filling loading of 1-5 wt%.

**Fig. S6.** Attenuation constant (ɑ) and intrinsic impedance ratio (*Z*) of **a** RGO composites and **b** Fe/RGO-2 composites at a low filling loading of 1-5 wt%.

From the attenuation point of view, the attenuation constant ($\alpha$) can be expressed as follows and is an important factor to evaluate the dissipation effect for EMW [S1, S2].

$\alpha=\frac{\sqrt{2}\pi f}{c}\times\sqrt{\left( \mu"\varepsilon"-\mu'\varepsilon' \right)+\sqrt{\left( \mu"\varepsilon"-\mu'\varepsilon' \right)^{2}+\left( \varepsilon'\mu"+\varepsilon"\mu' \right)^{2}}}$ (1)

Based on formula (1), the larger the imaginary part of EM parameters of the material is, the larger $\alpha$ is obtained, meaning more heat energy consumption are converted easily. From Fig. S6, the attenuation performance gradually increases with the increase of RGO and Fe/RGO filling ratio. The impedance matching ratio (*Z*) of samples can be calculated as [S3]:

$Z=\left| \frac{Z_{in}}{Z_{0}} \right|=\left| \sqrt{\frac{\mu_{r}}{\varepsilon_{r}}} \right|$ (2)

$Z_{in}$ and $Z_{0}$ are intrinsic impedance matches of the absorbing material and vacuum, respectively. The *Z* value of the absorbing material is closer to 1, the better the impedance match, so that more electromagnetic waves enter the material to promote the absorption of electromagnetic energy. The *Z* values exhibit the opposite trend compared to $\alpha$. With the increase of the filling ratio, the *Z* values gradually decrease due to the increase of electrical conductivity.

**Fig. S7.** Structure of Fe (110)/graphene (001) system (showing top view **a** and side view **b**) and charge density difference **c**, with purple and gray balls representing Fe atom and C atom, red contour denotes electron depletion region and blue contour refers to electron accumulation region.

**Fig. S8.** Microstructural characterizations of graphene and Fe/graphene. **a** Raman spectra, **b** TEM image, **c-f** SAED patterns and **g** AFM topography signals of graphene. **h** TEM and **i** SAED pattern of Fe/graphene.

**Fig. S9. a** µ' and **b** µ" of RGO, Fe/RGO-1, Fe/RGO-2, Fe/RGO-3 and Fe/RGO-4. **c** Magnetic hysteresis loops of Fe, Fe_2_O_3_, Fe/RGO-4.

Due to the low Fe content (0.32 ~1.22 wt%) in Fe/RGO composites, the u' and u" values of all samples are ≈1 and ≈0 (Fig. S9), and the saturation magnetization (Ms) value of Fe/RGO-4 was only 0.8 % of Fe (1.40 emu/g, 169.08 emu/g), indicating that Fe has no obvious magnetic loss at the measured frequency in the alternating EM field.

**Fig. S10.** The RL values versus frequency and thickness for **a-c** RGO, **d-f** Fe/RGO-1, **g-i** Fe/RGO-2, **j-l** Fe/RGO-3 and **m-o** Fe/RGO-4. The region confined in the black lines corresponds to RL≤−10 dB.

**Fig. S11.** RL values, simulated and experimental values of matching thicknesses under λ/4 condition, impedance matching values for a RGO, b Fe/RGO-2, c Fe/RGO-3 over 2-18 GHz.

**Fig. S12.** Cole-Cole curves of ε'-ε" and ε'- ε"_p_/f of **a** RGO, **b** Fe/RGO-1, **c** Fe/RGO-2, **d** Fe/RGO-3 and **e** Fe/RGO-4.

Based on the Debye relaxation theory, the existence of polarization can be proved by the Cole-Cole semicircle, each semicircle of Cole-Cole expresses a Debye relaxation process [S4]. The ԑ' and ε" can be expressed as follows:

$\varepsilon\text{' = }\frac{\text{(}\varepsilon_{\text{s}}\text{-}\varepsilon_{\text{∞}}\text{)}}{\text{1}\text{+}{(2\pi f)}^{\text{2}}\text{τ}^{\text{2}}}\text{+ }\varepsilon_{\text{∞}}$ (3)

$\varepsilon\text{" = }\varepsilon_{\text{p}}\text{" +}\varepsilon_{\text{c}}\text{" =}\frac{\varepsilon_{\text{s}}\text{-}\varepsilon_{\text{∞}}}{\text{1}\text{+}{(2\pi f)}^{\text{2}}\text{τ}^{\text{2}}}2\pi f\text{τ+}\frac{\text{σ}}{2\pi f\varepsilon_{\text{0}}}$ (4)

$\left( \varepsilon^{'}-\frac{\varepsilon_{s}+\varepsilon_{\infty}}{2} \right)^{2}+\left( \varepsilon" \right)^{2}=\left( \frac{\varepsilon_{s}-\varepsilon_{\infty}}{2} \right)^{2}$ (5)

where $\varepsilon_{\text{s}}$ is the static permittivity, $\varepsilon_{\infty}$ is the relative dielectric constant, τ is the relaxation time, respectively. To extract the τ of each polarization behavior, equation (3) and (4) can be further reduced to equation (6):

$\varepsilon\text{'(}f\text{) =}\frac{\text{1}}{2\pi f\text{τ}}\text{ }\varepsilon_{\text{p}}\text{"(}f\text{)+ }\varepsilon_{\text{∞}}$ (6)

According to equation (6), each straight line in the plots of $\varepsilon\text{'}$ versus $\varepsilon_{\text{p}}\text{"/f}$ represents a kind of polarization behavior with different relaxation times [S5]. Fig. S12 shows the $\varepsilon$'-$\varepsilon$" and $\varepsilon\text{'}$-$\varepsilon_{\text{p}}\text{"(}f\text{)}$ curves of RGO, Fe/RGO-1, Fe/RGO-2, Fe/RGO-3 and Fe/RGO-4, it can be seen that the number of semicircles increases with the increase of Fe, indicating an enhanced relaxation process compared with GO. Fe/RGO-2 and Fe/RGO-3 have more polarization relaxation processes. The relaxation times are listed in Table S1.

**Fig. S13.** The tanδ_ε_ **a**, α **b**, and *Z* **c** of RGO, Fe/RGO-1, Fe/RGO-2, Fe/RGO-3 and Fe/RGO-4.

**Fig. S14.** The electromagnetic parameters **a-d** and RL values **e-h** of Fe/RGO-1', Fe/RGO-2', Fe/RGO-3' and Fe/RGO-4'.

**
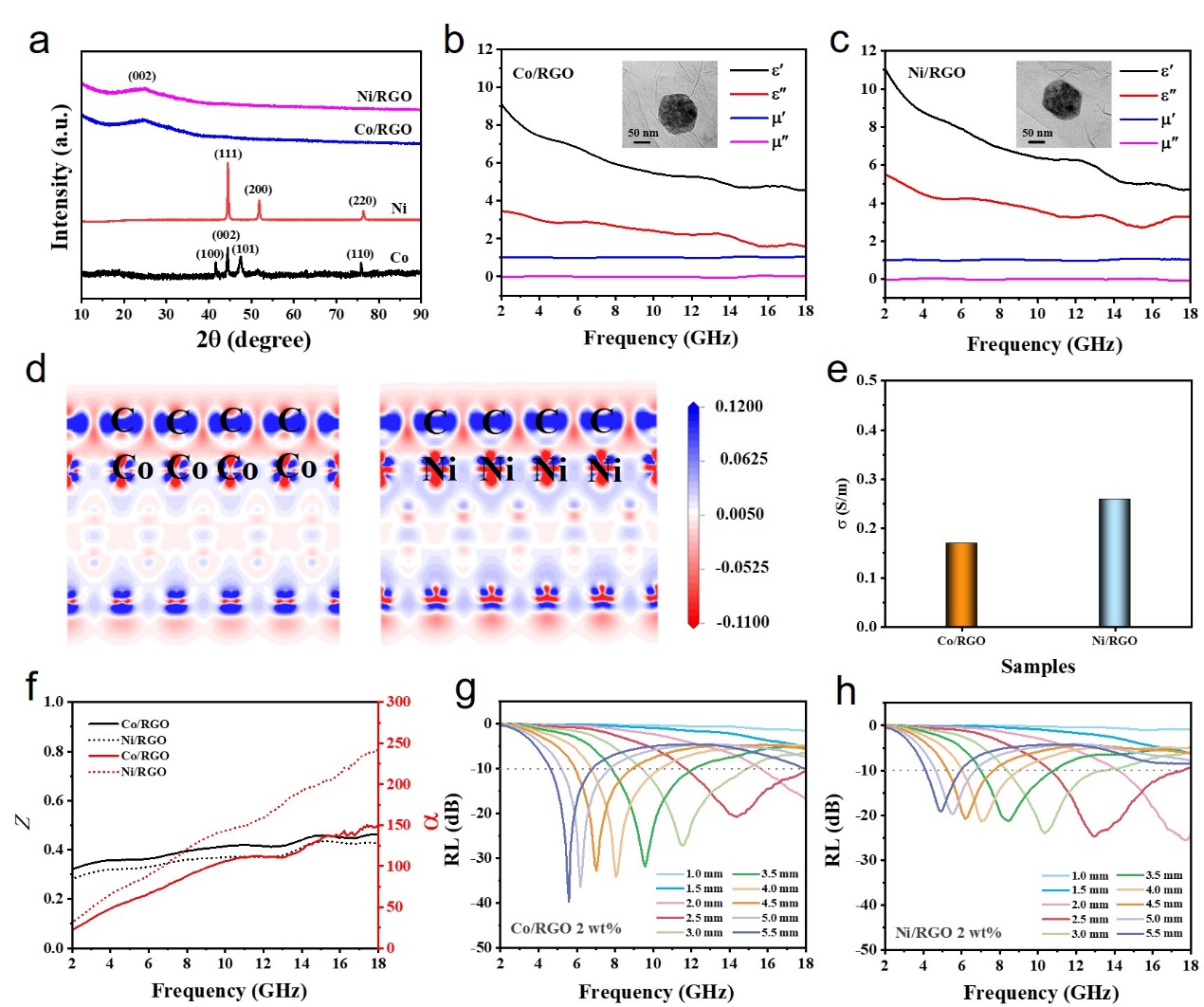
**
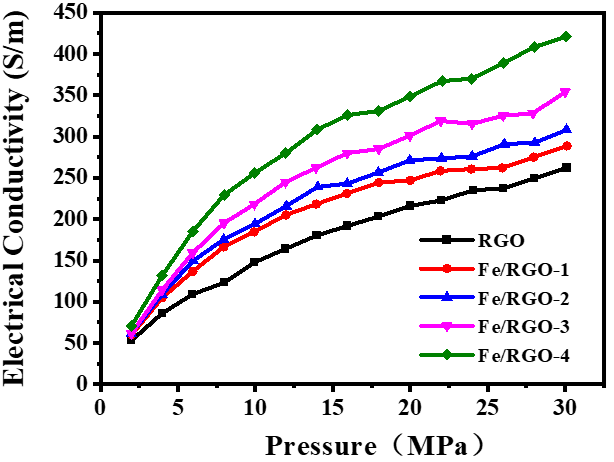
**Fig. S15.** The electrical conductivity of RGO, Fe/RGO-1, Fe/RGO-2, Fe/RGO-3 and Fe/RGO-4 powders.

**Fig. S16.** XRD patterns of Co, Ni, Co/RGO, Ni/RGO **a**. Eelectromagnetic parameters (ε', ε", μ' and μ") of Co/RGO and Ni/RGO composites with 2 wt% loading **b, c**. Charge density difference plots of Co (002)/graphene (001) and Ni (111)/graphene (001). The positive and negative charges are shown in blue and red (isovalue: 0.1) **d**. The σ **e**, *Z* and α **f** of Co/RGO and Ni/RGO. RL values with different thickness of Co/RGO **g** and Ni/RGO with 2 wt% loading **h**.


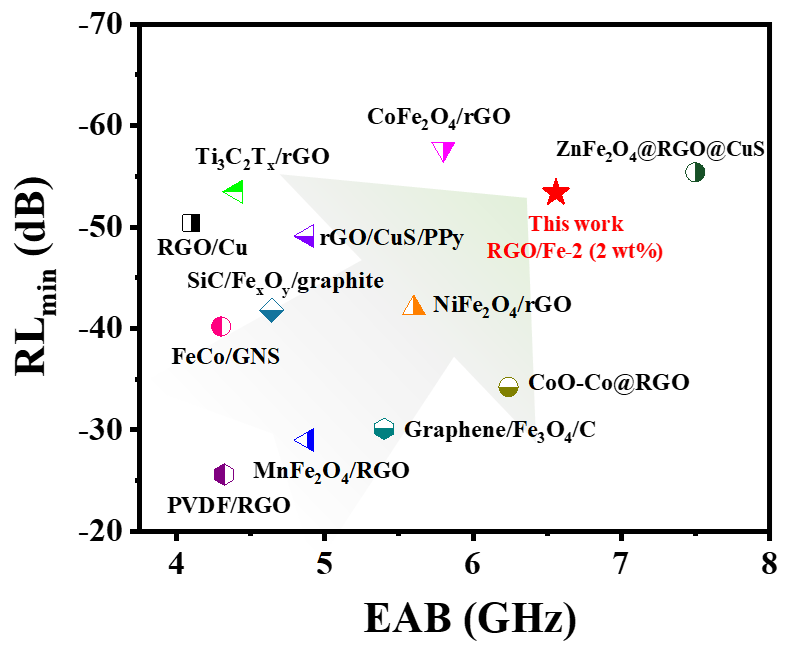
**Fig. S17.** Comparison of some typical RGO-based composites reported in the recent literature.

**Table S1** Parameters of the local dielectric detection

| **Parameters** | **value** |
| --- | --- |
| Quality factor (*Q*) | 181 |
| Elastic coefficient (*k*) | 3 N/m |
| AC voltage (*V*_AC_) | 10 V |
| Lift height (*z*) | 15 nm |

**Table S2** Relaxation times of the RGO, Fe/RGO-1, Fe/RGO-2, Fe/RGO-3 and Fe/RGO-4

| **Samples** | **Frequency (GHz)** | **τ (s)** |
| --- | --- | --- |
| RGO | 2 - 4.32 | 6.21×10^-11^ |
|  | 4.32 - 9.76 | 1.59×10^-11^ |
|  | 9.76 - 18 | 2.45×10^-12^ |
| Fe/RGO-1 | 2 - 4.79 | 8.22×10^-11^ |
|  | 4.79 - 11.27 | 2.142×10^-11^ |
|  | 11.27 - 14.52 | 4.17×10^-12^ |
|  | 14.52 - 18 | 3.54×10^-12^ |
| Fe/RGO-2 | 2 - 3.56 | 9.43×10^-11^ |
|  | 3.56 - 4.78 | 7.24×10^-11^ |
|  | 4.78 - 7.67 | 2.89×10^-11^ |
|  | 7.67 - 9.78 | 1.73×10^-11^ |
|  | 9.78 - 14.94 | 8.85×10^-12^ |
|  | 14.94 - 18 | 8.18×10^-12^ |
| Fe/RGO-3 | 2 - 2.52 | 9.36×10^-11^ |
|  | 2.52 - 3.74 | 6.41×10^-11^ |
|  | 3.74 - 5.67 | 5.37×10^-11^ |
|  | 5.67 - 8.73 | 1.77×10^-11^ |
|  | 8.73 - 14.85 | 7.15×10^-12^ |
|  | 14.85 - 18 | 3.72×10^-12^ |
| Fe/RGO-4 | 2 - 4.12 | 2.68×10^-11^ |
|  | 4.12 - 5.79 | 1.41×10^-11^ |
|  | 5.79 - 10.54 | 7.58×10^-12^ |
|  | 10.54 - 18 | 4.14×10^-12^ |

**Table S3** The EMA performance in 2-18 GHz of typical absorbers

| **Filler** | **Mass Ratio (wt%)** | **Thickness (mm)** | **RL_min_ (dB)** | **EAB**  **(GHz)** | **Refs.** |
| --- | --- | --- | --- | --- | --- |
| RGO/Cu | 8 | 5 | -50.4 | 4.1 | [S6] |
| FeCo/GNS | 50 | 2.5 | -40.2 | 4.3 | [S7] |
| NiFe_2_O_4_/rGO | 70 | 5 | -42 | 5.6 | [S8] |
| CoFe_2_O_4_/rGO | 50 | 2.8 | -57.7 | 5.8 | [S9] |
| Ti_3_C_2_T_x_/rGO | 5 | 2.2 | -53.49 | 4.4 | [S10] |
| MnFe_2_O_4_/RGO | 10 | 3.0 | -29.0 | 4.88 | [S11] |
| rGO/CuS/PPy | 10 | 4 | -49.11 | 4.88 | [S12] |
| PVDF/RGO | 3 | 4 | -25.6 | 4.32 | [S13] |
| CoO-Co@RGO | 10 | 2.1 | -34.22 | 6.24 | [S14] |
| Graphene/Fe_3_O_4_/C | 25 | 1.8 | -30.1 | 5.4 | [S15] |
| SiC/Fe_x_O_y_/graphite | 66.67 | 5.5 | -41.8 | 4.64 | [S16] |
| ZnFe_2_O_4_@RGO@CuS | 20 | 2.2 | -55.4 | 7.5 | [S17] |
| **Fe/RGO-2** | **2** | **2.45** | **-53.38** | **6.56** | **This work** |
|  | **2** | **2.62** | **-29.33** | **7.52** |  |

**Supplementary References**

1. X. Liu, Y. Duan, Y. Guo, H. Pang, Z. Li et al., Microstructure design of high-entropy alloys through a multistage mechanical alloying strategy for temperature-stable megahertz electromagnetic absorption. Nano-Micro Lett. **14**(1), 142 (2022). <https://doi.org/10.1007/s40820-022-00886-6>
2. L. Zhang, X. Zhang, G. Zhang, Z. Zhang, S. Liu et al., Investigation on the optimization, design and microwave absorption properties of reduced graphene oxide/tetrapod-like ZnO composites. RSC Adv. **5**(14), 10197-10203 (2015). <https://doi.org/10.1039/c4ra12591f>
3. K. Zhang, J. Chen, S. Yue, H. Zhang, C. Meng et al., Facile synthesis of core-shell CI/SiO_2_ decorated RGO sheets composite for excellent electromagnetic wave absorption performance covering the whole x-band. Compos. Part A-Appl. S. **130**, 105755 (2020). <https://doi.org/10.1016/j.compositesa.2019.105755>
4. W. Liu, S. Tan, Z. Yang, G. Ji, Hollow graphite spheres embedded in porous amorphous carbon matrices as lightweight and low-frequency microwave absorbing material through modulating dielectric loss. Carbon **138**, 143–153 (2018) <https://doi.org/10.1016/j.carbon.2018.06.009>
5. X. Zhang, P. Guan, X. Dong, Multidielectric polarizations in the core/shell Co/graphite nanoparticles. Appl. Phys. Lett. **96**, 223111 (2010). <https://doi.org/10.1063/1.3446868>
6. H. Zhang, X. Tian, X. Zhang, S. Li, Y. Shen et al., Preparation and electromagnetic wave absorption of rgo/Cu nanocomposite. J. Phys. Chem. A **91**(9), 1771-1774 (2017). <https://doi.org/10.1134/s0036024417090126>
7. X. Li, J. Feng, Y. Du, J. Bai, H. Fan et al., One-pot synthesis of CoFe_2_O_4_/graphene oxide hybrids and their conversion into FeCo/graphene hybrids for lightweight and highly efficient microwave absorber. J. Mater. Chem. A **3**(10), 5535-5546 (2015). <https://doi.org/10.1039/c4ta05718j>
8. J. He, X. Wang, Y. Zhang, M. Cao, Small magnetic nanoparticles decorating reduced graphene oxides to tune the electromagnetic attenuation capacity. J. Mater. Chem. C **4**(29), 7130-7140 (2016). <https://doi.org/10.1039/c6tc02020h>
9. Y. Liu, Z. Chen, Y. Zhang, R. Feng, X. Chen et al., Broadband and lightweight microwave absorber constructed by in situ growth of hierarchical CoFe_2_O_4_/reduced graphene oxide porous nanocomposites. ACS Appl. Mater. Interfaces **10**(16), 13860-13868 (2018). <https://doi.org/10.1021/acsami.8b02137>
10. S. Shang, N. Zhao, Y. Chen, X. Wang, F. Hu et al., Ti_3_C_2_T_x_/rgo aerogel towards high electromagnetic wave absorption and thermal resistance. CrystEngComm **24**(25), 4556-4563 (2022). <https://doi.org/10.1039/d2ce00578f>
11. F. Wen, H. Hou, J. Xiang, X. Zhang, Z. Su et al., Fabrication of carbon encapsulated Co_3_O_4_ nanoparticles embedded in porous graphitic carbon nanosheets for microwave absorber. Carbon **89**, 372-377 (2015). <https://doi.org/10.1016/j.carbon.2015.03.057>
12. B. Zhang, S. Lin, J. Zhang, X. Li, X. Sun, Facile synthesis of sandwich-like rGO/CuS/Polypyrrole nanoarchitectures for efficient electromagnetic absorption. Materials **13**, 446 (2020). <https://doi.org/10.3390/ma13020446>
13. X. Zhang, G. Wang, W. Cao, Y. Wei, M. Cao et al., Fabrication of multi-functional PVDF/RGO composites via a simple thermal reduction process and their enhanced electromagnetic wave absorption and dielectric properties. RSC Adv. **4**(38), 19594-19601 (2014). <https://doi.org/10.1039/c4ra02040e>
14. Y. Meng, S. Lu, Y. Wu, Z. Zi, Y. Ma et al., Efficient electromagnetic wave absorption of porous CoO–Co@RGO composites with optimized impedance matching derived from metal-organic frameworks. Nano **15**(08), 2050104 (2020). <https://doi.org/10.1142/s1793292020501040>
15. Y. Huang, L. Wang, X. Sun, Sandwich-structured graphene@Fe_3_O_4_@carbon nanocomposites with enhanced electromagnetic absorption properties. Mater. Lett. **144**, 26-29 (2015). <https://doi.org/10.1016/j.matlet.2015.01.015>
16. R. Meng, T. Zhang, P. Jiao, M. Zhang, X. Huang et al., Facile fabrication of SiC/Fe_x_O_y_ embellished graphite layers with enhanced electromagnetic wave absorption. J. Alloys Compd. **798**, 386-393 (2019). <https://doi.org/10.1016/j.jallcom.2019.05.210>
17. Y. Wang, X. Gao, X. Wu, W. Zhang, Q. Wang et al., Hierarchical ZnFe_2_O_4_@RGO@CuS composite: strong absorption and wide-frequency absorption properties. Ceram. Int. **44**(8), 9816-9822 (2018). <https://doi.org/10.1016/j.ceramint.2018.02.220>
